# A Review of the Genus *Oecetis* (Trichoptera: Leptoceridae) in the Northeastern Region of Brazil with the Description of 5 New Species

**DOI:** 10.1371/journal.pone.0127357

**Published:** 2015-06-10

**Authors:** Fabio Batagini Quinteiro, Adolfo Ricardo Calor

**Affiliations:** 1 Faculdade de Filosofia, Ciências e Letras de Ribeirão Preto, Universidade de São Paulo, Ribeirão Preto, São Paulo, Brazil; 2 Instituto de Biologia, Universidade Federal da Bahia, Salvador, Bahia, Brazil; Louisiana State University, UNITED STATES

## Abstract

Within Leptoceridae, the genus *Oecetis* contains about 500 species around the world, including 53 in the Neotropics. In Brazil, there are 15 recorded species of *Oecetis*. These species were described over several decades by numerous authors with the results that descriptions are not comparable and diagnoses are incomplete. Also, the apparently unbranched M vein, in the forewing, a diagnostic character for *Oecetis* pointed by McLachlan, is controversial and no consensus has been reached about its homology. Additionally, the only revision for the genus was never published; thus the information and proposed taxa are not available according to the International Code of Zoological Nomenclature. We analyzed specimens collected in the Brazilian Northeast Region and compared these with described species and literature descriptions and *Oecetis* from other regions. We provide herein the description of five new species, additional characters for diagnosing seven of the species recorded from Brazil, new distributional records, and a dichotomous key to the Brazilian species. Additionally, we contrast the two hypotheses of forewing M vein homology and support the unbranched hypothesis. In this way, we improve the knowledge of the genus in the Neotropics, making the species descriptions comparable in a way that facilitates species identification.

## Introduction

Leptoceridae is one of the three largest families of Trichoptera, with more than 2,000 described species [1]. The family is comprised of 48 extant genera, placed traditionally in two subfamilies, Triplectidinae Ulmer and Leptocerinae Leach, the former recorded from Australasian and Neotropical regions, while the latter has a cosmopolitan distribution [2]. Recently, Malm & Johanson [2] raised Grumichellini Morse, with Neotropical and Australian distribution [3], and Leptorussini Morse, with Australian distribution [3], to subfamily status, classifying the family into four subfamilies.

Within Leptocerinae, the genus *Oecetis* McLachlan [4] contains about 500 species around the world [5], including 53 in Neotropics [5] and 31 in South America [5–8]. The genus was erected by McLachlan from *Setodes* Rambur by the apparently unbranched median vein of the forewing [9]. Although McLachlan did not establish a type species, Ross [10] subsequently selected *O*. *ochracea* [11].

The majority of original descriptions of *Oecetis* species are not detailed, and include few diagnostic characters. The latest comprehensive treatment of the genus, including the Brazilian species, was implemented by Chen [9], in his unpublished PhD thesis. He proposed a classification of the genus into four subgenera: *Pleurograpta*, *Pseudosetodes*, *Oecetis*, and *Quaria*. The two former names were first proposed as genera by Wallengren [12] and Ulmer [13], respectively, but trated as synonyms by Fischer [14] and Flint [15], respectively. The name *Quaria* was proposed by Milne [16] as a subgenus of *Oecetis*. The subgenera recognized in Chen’s unpublished work [9] are considered “species groups” here. These four species groups show very distinctive male genitalic characteristics and are easily recognizable. According to Chen [9], *Pseudosetodes* is the only species group that has a symmetrical phallus without phallic spines. *Oecetis* has only one phallic spine, and an asymmetrical phallus. *Pleurograpta* has a short tergum IX and a long sternum IX, and males of *Quaria* are easily distinguished by the presence of a pair of prominent dorsolateral processes on tergum IX.

In Brazil, 15 species of *Oecetis* are recorded (with their respective distributions): *O*. *amazonica* (Banks, 1924) [17] (Amazonas and Mato Grosso do Sul States), *O*. *angelae* Henriques-Oliveira, Dumas & Nessimian 2014 [8] (Mato Grosso do Sul State), *O*. *connata* Flint, 1974 [18] (Amazonas, Bahia, Pará, Piauí, Rio de Janeiro and São Paulo States), *O*. *danielae* Henriques-Oliveira, Dumas & Nessimian 2014 [8] (Amazonas State), *O*. *doesburgi* Flint 1974 [18] (Amazonas State), *O*. *dominguezi* Rueda-Martín, Gibon & Molina 2011 [6] (Mato Grosso do Sul State), *O*. *excisa* Ulmer, 1907 [19] (Bahia, Ceará, Mato Grosso do Sul and Pará States), *O*. *fibra* Chen & Morse *in* Quinteiro & Calor, 2012 [7] (Espírito Santo, Paraná, Rio de Janeiro, Santa Catarina and São Paulo States), *O*. *iara* Henriques-Oliveira, Dumas & Nessimian 2014 [8] (Paraná State), *O*. *iguazu* Flint, 1983 [20] (Bahia, Espírito Santo, Minas Gerais, Paraná, Rio de Janeiro, Santa Catarina, and São Paulo States), *O*. *incospicua* (Walker, 1852) [21] (Amazonas, Bahia, Minas Gerais, Paraíba, Paraná, Piauí and Paraná States), *O*. *knutsoni* Flint 1981 [22] (Amazonas State), *O*. *paranensis* Flint, 1982 [23] (Bahia, Mato Grosso do Sul and Minas Gerais States), *O*. *punctipennis* (Ulmer, 1905) [13] (Bahia, Ceará, Minas Gerais, Pará, Pernambuco, Rio de Janeiro and São Paulo States) and *O*. *rafaeli* Flint, 1991 [24] (Roraima State) ([7,8,25–28]). It is evident that some regions are less well known than others; lack of representation of the Northeast region has already been noted [28–30].

Concentrating our efforts in the Northeast region, to minimize this disparity, we provide, herein, a review of Brazilian *Oecetis* species, describe five new species, and provide a synopsis of the previously described *Oecetis*, improving the original descriptions with new characters and illustrations. We also present a dichotomous key for all the known Brazilian species.

Additionally, it is known that some *Oecetis* species are difficult to diagnose [31]. In Brazil, we have records of four of these cited species: *Oecetis excisa* and *O*. *inconspicua*, which are stated as possible synonyms by some authors (*e*.*g*., [6]; [32]); and *O*. *iguazu* and *O*. *punctipennis*. In order to explore additional morphological characters for improving the diagnosis of these species, they were examined by using a Scanning Electron Microscope, and the micrographs and discussion of results are herein presented.

Finally, in the literature, there are two divergent interpretations of *Oecetis* forewing venational homology: M vein in forewing unbranched *versus* M vein branched into M_1+2_ and M_3+4_. Based on a review of the literature and observation of specimens, our evidence support the first interpretation: M is unbranched.

In this way we improve the knowledge of caddisflies in Brazil, by revealing unknown diversity and distributional patterns, solving a long standing problem in caddisfly literature and propose a standard nomenclature and homology hypothesis for the genus wings.

## Material and Methods

Specimens were collected using light traps and pan light traps [33] with incandescent and fluorescent lamps placed next to water bodies. The collecting trips were made with support of Dr. Vitor Becker (Reserve Serra Bonita) and Aquatic Entomology Lab staff (LEAq-UFBA). In addition, Malaise traps were also used over low order streams. The specimens collected were stored in 80% alcohol or pinned. Genitalia were removed, together with 4 to 5 abdominal segments, cleared for identification using 85% lactic acid solution [34] or 10% KOH and stored in microvials containing glycerin [35,36]. Illustrations were made using a compound microscope with drawing tube attached. Improvements on illustrations were made using the software Adobe Illustrator CS 5. Morphological terminology followed Snodgrass [37] and Schmid [38], as implemented by Holzenthal [35] and Calor [39]. The lengths presented in the description sections are average lengths. The DELTA System [40–42] was used to produce the species descriptions and the dichotomous key.

Type material will be deposited at Museu de Zoologia, Universidade de São Paulo (MZUSP), Museu de Zoologia, Universidade Federal da Bahia (UFBA) and the University of Minnesota Insect Collection (UMSP), as indicated in material examined.

The specimens examined by Scanning Electron Microscope (SEM) had their genitalia cut off with a pair of iridectomy scissors, and were subsequently positioned on a carbon tape, metalized, and then the photos were taken in a JEOL JSM 6390LV microscope.

Acronyms for the Brazilian States are the following: Amazonas (AM), Bahia (BA), Ceará (CE), Espírito Santo (ES), Mato Grosso (MT), Minas Gerais (MG), Pará (PA), Paraíba (PB), Paraná (PR), Pernambuco (PE), Piauí (PI), Rio de Janeiro (RJ), Rio Grande do Norte (RN), Rondônia (RO), Roraima (RR), Santa Catarina (SC) and São Paulo (SP).

### Nomenclatural acts

The electronic edition of this article conforms to the requirements of the amended International Code of Zoological Nomenclature, and hence the new names contained herein are available under that Code from the electronic edition of this article. This published work and the nomenclatural acts it contains have been registered in ZooBank, the online registration system for the ICZN. The ZooBank LSIDs (Life Science Identifiers) can be resolved and the associated information viewed through any standard web browser by appending the LSID to the prefix "http://zoobank.org/". The LSID for this publication is: urn:lsid:zoobank.org:pub:01A88E9B-558A-4469-83E7-C89406BCD5E0. The electronic edition of this work was published in a journal with an ISSN, and has been archived and is available from the following digital repositories: PubMed Central and LOCKSS.

## Taxonomy

***Oecetis acanthostema* sp. nov.** urn:lsid:zoobank.org:act:E6612BEA-6738-4836-AFDB-240164239E68

(Fig 1)

**Fig 1:**
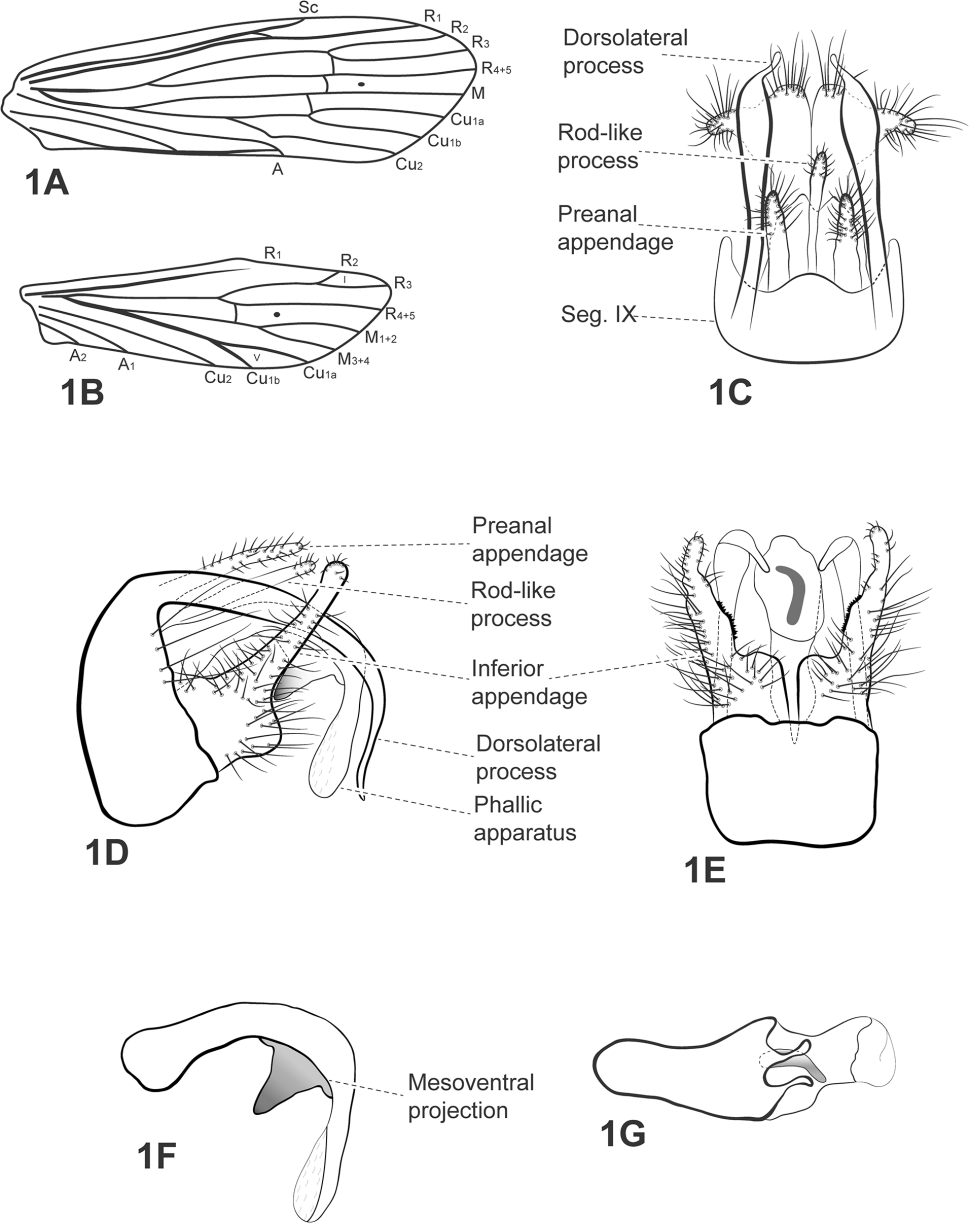
*Oecetis acanthostema* sp. nov. A, forewing. B, hind wing. C, male genitalia, dorsal view. D, male genitalia, lateral view. E, male genitalia, ventral view. F, phallic apparatus, lateral view. G, phallic apparatus, dorsal view. Abbreviation of the wing structures: Sc, subcosta; R, radius; M, medius; A, anal vein; I, fork 1; V, fork 5.

*Diagnosis*: The new species presents a pair of prominent dorsolateral processes on tergum IX of males, as in species of the *Quaria* species group (*sensu* Chen [9]), but can be distinguished from others of the group by the following characters: presence of a sclerotized projection on the mesoventral region of the phallic apparatus, and the distal 1/3 of the dorsolateral process enlarged in width and flattened dorsoventrally; this process is cylindrical in other species of the species group.

*Male*: body length 5 mm (n = 12). Forewing length 5.6 mm (n = 12).

Head: color pale yellowish-brown (alcohol). Antennae very long, about 3.5 length of forewings. Maxillary palps yellow, densely covered with setae, 5-segmented, all segments subequal in length and width. Labial palps pale yellow, 3-segmented.

Thorax: Pterothorax yellowish brown in dorsal region and pale yellow in lateral and ventral regions. Forewings hyaline, yellowish brown, with 3 dark transversal bands (not visible in Fig 1A and 1B) over crossveins *s*, *r–m* and *m–cu*; wing vein pattern as in Fig 1A and 1B. Hind wings each with row of setae along posterior margin; forks I and V present (Fig 1B). Legs yellowish-brown. Mid leg each with longitudinal row of spines from distal half of femur to the first tarsal segment. Tibial spur formula 1,2,2. Apical spur of fore tibia very small.

*Genitalia*: Dorsolateral processes of segment IX slender, bent ventrad, tapering posteriorly and extending beyond length of phallic apparatus (Fig 1C); 2/3^rd^ of process enlarged in width, flattened, dorsal view (Fig 1C); four long setae on posterior margin of segment IX, near base of dorsolateral processes (Fig 1D). Preanal appendage long, digitate with short setae. Rod-like mesodorsal process above tergum X present, with few short setae on apex (Fig 1C). Tergum X membranous, divided medially, forming two lobes, broad basally and acute apically, with shallow cleft between them in dorsal view (Fig 1C). Inferior appendage 1-segmented, broad basally, covered with setae over its base and mid portion; upper portion digitate, covered with small setae on top; apex rounded in lateral view (Fig 1D); presence of small spine-like setae on inner portion in ventral view (Fig 1E). Phallic apparatus long, bent ventrad, with spine-like projection on ventral mid portion, sclerotized on apex (Fig 1F); asymmetrical, bearing two small, digitate mesolateral projections in dorsal view (Fig 1G).

*Holotype* (*male in alcohol; MZUSP*): **Brazil**, **Bahia**, Senhor do Bonfim, Serra Santana, 28.xi.2006 (Souza, Monteiro, Alvim, Rocca).

*Paratypes*: **Brazil**, **Bahia**, Senhor do Bonfim, Serra Santana, 28.xi.2006 (Souza, Monteiro, Alvim, Rocca)– 3 males (alcohol; UFBA, UMSP); same data except 21–22.vii.2009 (Zacca, T., Lopes, P., Mota, E., Menezes, E.)– 1 male (alcohol; MZUSP); Pindobaçú, Cachoeira da Fumaça, 10°28’43”S, 40°12’27.6”W, 16.xii.2009 (Zacca, T.)– 1 male (alcohol; UFBA); same data except 17.xii.2009 – 3 males, 2 females (alcohol; MZUSP, UMSP); Palmeiras, Capão, Pousada Capão, 12°37’21.7”S, 41°29’11.7”W, 938m, 21.vi.2011 (Calor, A.R., Camelier, P., Burger, R.)– 1 male, 1 female (alcohol; MZUSP).

*Distribution*: Brazil (BA).

*Etymology*: from Greek *acanthus* = spine; *stemum* = penis. The species name is in reference to the presence of a spine-like projection on the mesoventral region of the phallic apparatus.

*Taxonomic remarks*: *Oecetis acanthostema* sp. nov. is clearly a member of the *Quaria* species group due to the presence of two long dorsolateral processes on segment IX of the male genitalia. The new species has two characteristics that no other species in the genus presents: presence of a spine-like process on the mesoventral portion of the phallic apparatus, and distal 1/3 of the dorsolateral processes enlarged in width and flattened dorsoventrally, like the neck of a cobra. This species is similar to *Oecetis fibra*, because of the shared presence of a long phallic apparatus, short and digitate preanal appendage and similar shape of the inferior appendage, but *O*. *acanthostema* sp. nov. has an inferior appendage with a broad base. The shape of tergum X is digitate apically on *O*. *fibra*, while in the new species tergum X is similar to *O*. *rafaeli* [tergum X “divided into long, slender, lateral lobes, pointed apicad and overlaying phallic apparratus dorsolaterally”, in original description of Flint ([24], p.74). However, *O*. *rafaeli* has short and straight dorsolateral processes and no rod-like process above tergum X.

***Oecetis martinae* sp. nov.** urn:lsid:zoobank.org:act:B464EF98-7FBB-4FC4-AB7F-EBFDB1E1C0A5

(Fig 2)

**Fig 2:**
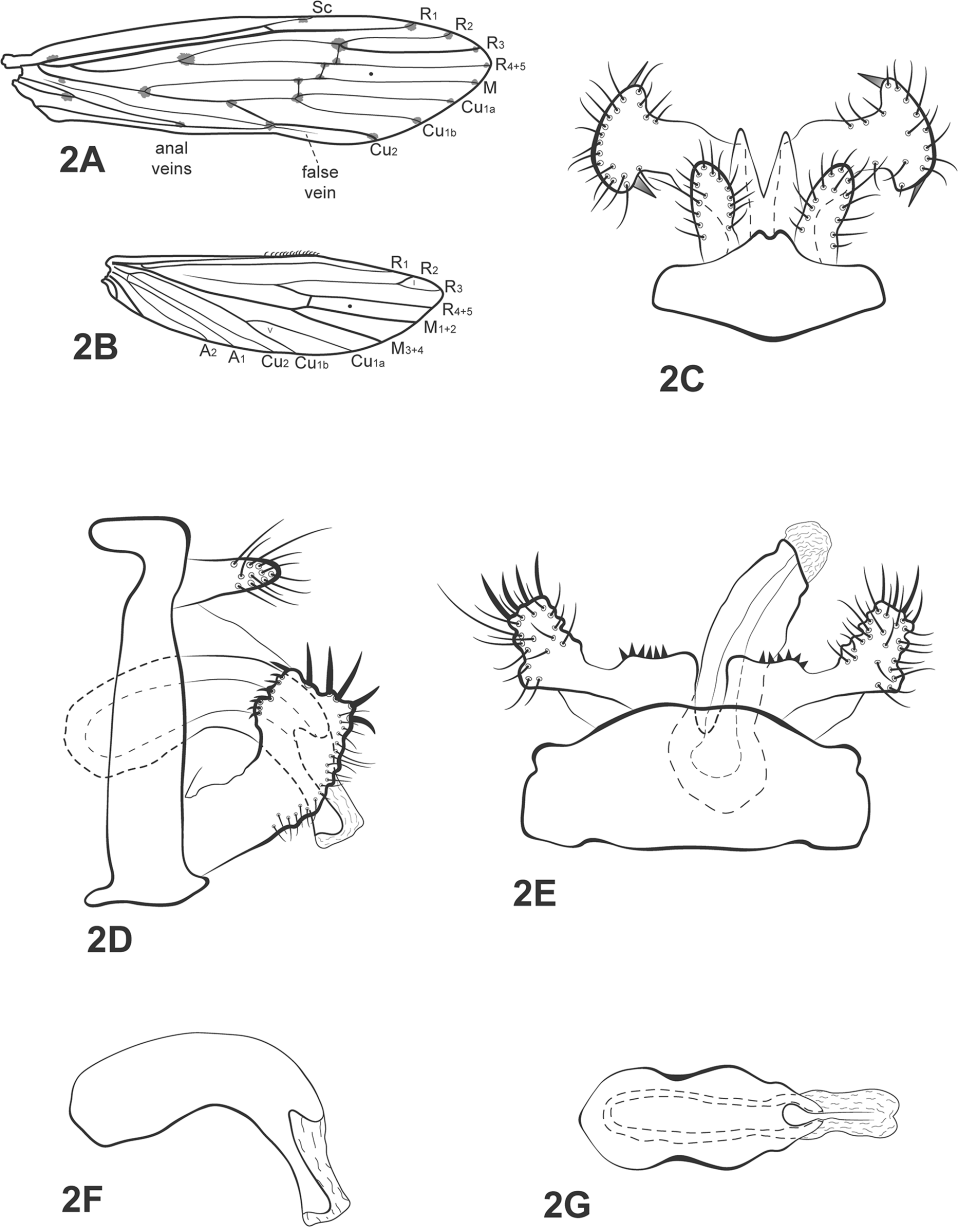
*Oecetis martinae* sp. nov. A, forewing. B, hind wing. C, male genitalia, dorsal view. D, male genitalia, lateral view. E, male genitalia, ventral view. F, phallic apparatus, lateral view. G, phallic apparatus, dorsal view. Abbreviation of the wing structures: Sc, subcosta; R, radius; M, medius; A, anal vein; I, fork 1; V, fork 5.

*Diagnosis*: This species is similar to *Oecetis chipiriri* Martin, Gibon & Molina [6], *Oecetis knutsoni* Flint [22], and *O*. *punctata* (Navás) [43]. This new species has a quadrate inferior appendage, while *O*. *chipiriri* and *O punctata* have a rounded one. The inferior appendages of *O*. *knutsoni* and *O punctata* have small digitate projections on top bearing the thick setae, and a mesoventral process on the inferior appendages, both of which are absent in the new species. Moreover, *O*. *knutsoni* and *O*. *punctata* have a short, symmetrical phallic apparatus, with a U-shaped mesodorsal portion, while the new species has a long, asymmetrical, basally wide phallic apparatus, with a sinuous mesodorsal portion, which resembles the phallic apparatus of *O*. *chipiriri*. However, *O*. *chipiriri* has a rounded, thumb-like, inferior appendage, whereas *Oecetis martinae* sp. nov. has a quadrate one.

*Male*: body length 4.5 mm (n = 30). Forewing length 6.5 mm (n = 30).

Head: Pale yellow (alcohol). Antennae long, about 3 times length of forewings. Maxillary palps yellow, 5-segmented, all segments subequal in length and width, densely covered with setae. Labial palps pale yellow, 3-segmented.

Thorax: Pterothorax yellow in dorsal and pale yellow in lateral and ventral regions. Forewings hyaline, yellow with brownish dots over end of veins, forks, and junctions; dark bands over crossveins absent; wing vein pattern as in Fig 2A and 2B; crossvein *m-cu* reaching Cu_1a_ (after ramification of Cu into Cu_1a_ and Cu_1b_). Hind wings with basal brush; forks I and V present (Fig 2B). Legs yellowish-brown, each with row of spines on distal half of femur, all along tibia and first tarsal segment. Tibial spur formula 1,2,2; apical spur of fore tibia very small.

*Genitalia*: Segment IX annular and short (Fig 2D). Preanal appendage slightly longer than wide, ovoid, apex covered with setae (Fig 2C and 2D).Tergum X membranous, divided mesally, forming two lobes, broad basally, tapering apically, with V-shaped incision in dorsal view (Fig 2C). Inferior appendage 1-segmented, broad basally; dorsal lobe absent; ventral lobe absent; distal portion broad, quadrate, setose, with 5 thick setae on top and smaller ones next to those (Fig 2D); row of spine like setae present on inner surface (Fig 2E). Phallic apparatus slightly asymmetrical in dorsal view (Fig 2G); long, broad basally, bent ventrad, membranous apicad; apex quadrate, with sinuous projecting mesodorsal portion, in lateral view (Fig 2F).

*Holotype* (*male in alcohol; MZUSP*): **Brazil**, **Bahia**, Wenceslau Guimarães, Estação Ecológica Estadual Wenceslau Guimarães, Riacho Serra Grande, near Station headquaters, 13°35’43”S, 38°43’12”W, 08.x.2010, pan light trap U.V./white lights (Calor, A.R., Quinteiro, F.B., & col.).

*Paratypes*: **Brazil**, **Bahia**, Wenceslau Guimarães, Estação Ecológica Estadual Wenceslau Guimarães, Riacho Serra Grande, near Station headquaters, 13°35’43”S, 38°43’12”W, 08.x.2010, pan light trap U.V./white lights (Calor, A.R., Quinteiro, F.B., & col.)– 2 males, 2 females (alcohol; UFBA); same data except 27.x.2008 (Calor, A.R., Mariano, R., Mateus, S.)– 1 male (pinned; UFBA); 13°35’42”S, 39°43’12”W (different stream), 561m, 07.x.2010, pan light trap U.V./white lights (Calor, A.R., Quinteiro, F.B., & col.)– 2 males (alcohol; UMSP); same data except 09.x.2010 – 1 male, 1 female (alcohol; MZUSP), 1 male (pinned; UFBA); same data except 10.x.2010 – 27 males, 1 female (alcohol; UFBA); same data except upstream waterfall—2 males (alcohol; UMSP); Senhor do Bonfim, Serra Santana, light trap, 01.x.2005 (Almeida, Alvim)– 1 male (alcohol; MZUSP); Santa Teresinha, Serra da Jibóia, Torres stream, 06.xi.2010, light trap U.V./white lights (Calor, A.R., Quinteiro, F.B., Mariano, R., França, D., Costa, A.M.)– 1 male (alcohol; MZUSP); Pedra Branca, Serra da Jibóia, Lajedo waterfall, 12°51’00”S, 39°28’48”W, 678m, 09.vii.2008, light trap, U.V./white lights (Calor, A.R., Lecci, L.S., Pinho, L.C., Moretto, R.A)– 1 male (pinned; UMSP), 1 male (alcohol; UMSP); Camacan, Fazenda Altamira, 15°25’3.05”S, 39°33’9.89”W, 319m, 30.x.2008 (Calor, A., Mariano, R., Mateus, S.)– 1 male, 4 females (pinned; MZUSP), 2 males(alcohol; UFBA); 15°25’18.6”S, 39°33’59.3”W, 309m, 28.iii.2011 (Calor, A.R., Quinteiro, F.B., França, D., Barreto, H.)– 2 males (alcohol; UMSP); Fazenda Paris, Rio Branco do Sul, 03.iv.2011 (Quinteiro, F.B.,França, D., Barreto, H.)– 2 females; Camacan, RPPN Serra Bonita, stream after the dam supply, 15°25’16”S, 39°33’57”W, 300m, 05.vii.2008, light trap U.V./white lights (Calor, A.R., Lecci, L.S., Pinho, L.C., Moretto, R.A.)– 1 male (pinned; MZUSP); same data except, stream 3, path, 15°23’03”S, 39°34’00”W, 723m, 29.x.2008, light trap U.V./white lights (Calor, A.R., Mariano, R., Mateus, S.)– 2 males, 3 females (pinned; UMSP); same data except 01.viii.2008 (Calor, A.R., Lecci, L.S., Pinho, L.C., Moretto, R.A.)– 1 female (pinned; UFBA).

*Distribution*: Brazil (BA).

*Etymology*: this specific epithet honors Dr. Paola Rueda Martín (Facultad de Ciencias Naturales e Instituto Miguel Lillo, San Miguel de Tucumán, Argentina) who encouraged and provided helpful insights to this study.

*Taxonomic and collection remarks*: This new species is similar to *O*. *knutsoni*, *O*. *chipiriri*, and *O*. *punctata*. The major differences are in the inferior appendages. Although *O*. *martinae* sp. nov. has the thick spines on the inferior appendages, as do *O*. *knutsoni* and *O*. *punctata*, they differ significantly among them in details. *Oecetis knutsoni* has only three thick spines on the top of the inferior appendages and O. punctata has four of them. The new species has five thick spines and none of them bear digitate projections as in *O*. *knutsoni* and *O*. *punctata*. *Oecetis knutsoni* has a longer, but narrower inferior appendage and *O*. *punctata* has an upward curved process on the distal portion of the inferior appendage. The new species does not have this process and its appendage is not narrow, but quadrate as in *O*. *chipiriri*. The phallic apparatus is similar to *O*. *chipiriri* also, whereas *O*. *knutsoni* and *O*. *punctata* have shorter ones. The preanal appendages are very similar among the three species and the new one. The wing spots are also similar among this new species, *O*. *chipiriri* and *O*. *knutsoni*.

This species was collected at dusk, attracted by light, in low order coldwater streams, with rocks and rough sand in bed. The vegetation around was dense, composed of rainforest, where the light traps were set and the adults captured. They are typical from higher elevations (above 500m). However, in some collection sites, such as Santa Teresinha and Pedra Branca (BA), they can be found at lower elevations, where the streams are slightly different. These water bodies are shallow with intercalated channels and small pools. The vegetation is composed of smaller and sparser trees than the rainforest. The immature stages and the female of this species are unknown.

***Oecetis clavicornia* sp. nov.** urn:lsid:zoobank.org:act:031C365E-7603-4E58-957E-C267D8388133

(Fig 3)

**Fig 3:**
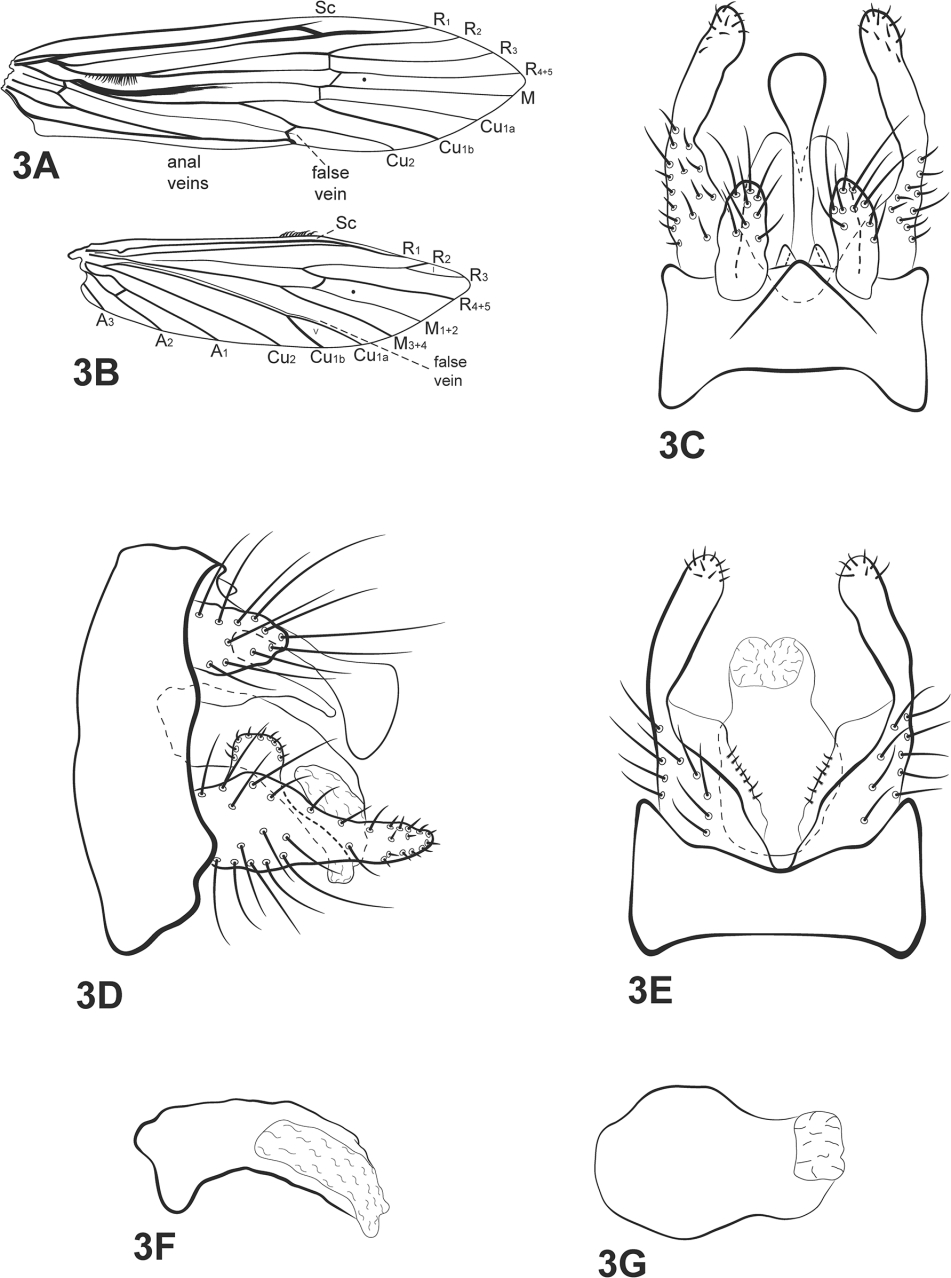
*Oecetis clavicornia* sp. nov. A, forewing. B, hind wing. C, male genitalia, dorsal view. D, male genitalia, lateral view. E, male genitalia, ventral view. F, phallic apparatus, lateral view. G, phallic apparatus, dorsal view. Abbreviation of the wing structures: Sc, subcosta; R, radius; M, medius; A, anal vein; I, fork 1; V, fork 5.

*Diagnosis*: The new species is similar to *Oecetis scoparia* Flint [18]. However, it has a small quadrate process on the dorsal region of the base of the inferior appendage, which is absent in *O*. *scoparia*. Moreover, the new species has a mesodorsal process above tergum X with a distinctly clavate apex, whereas *O*. *scoparia* has only a slightly clavate process, as described by Flint [18].

*Male*: body length 4.8 mm (n = 10). Forewing length 6.0 mm (n = 10).

Head: brown (alcohol). Antennae long, about 2.5 times length of forewings. Maxillary palps yellowish brown, 5-segmented, all segments subequal in length and width, densely covered with setae. Labial palps yellow, 3-segmented, first segment very small.

Thorax: Pterothorax yellowish brown in dorsal region and yellow in lateral and ventral regions. Forewings hyaline, yellowish brown with dark bands over cord; tuft of dark long setae present on base of M vein (Fig 3A); wing vein pattern as in Fig 3A and 3B; crossvein *m-cu* reaching Cu_1a_ (after branching of Cu into Cu_1a_ and Cu_1b_). Hind wings with basal brush; forks I and V present (Fig 3B). Legs yellowish brown, middle and posterior legs both with row of small spines on distal half of femur, all along tibia and tarsus. Tibial spur formula 0,2,2.

*Genitalia*: Segment IX annular and short. Preanal appendage longer than wide, ovoid, and covered with setae (Fig 3C and 3D). Tergum X membranous, divided mesally by V-shaped incision on distal 1/3 portion, forming two lobes, broad basally, rounded apically (Fig 3C); clavate process above tergum X present, longer than tergum X in lateral view (Fig 3D). Inferior appendage 1-segmented, broad basally, constricted mesally and rounded apically (Fig 3E); dorsal lobe present, quadrate, covered with small setae (Fig 3D); ventral lobe absent; row of spine like setae present on mesal surface (Fig 3E). Phallic apparatus asymmetrical, enlarged on mid portion in dorsal view (Fig 3G); in lateral view (Fig 3F), long, slightly widened basally, bent ventrad, membranous apically.

*Holotype* (*male in alcohol; MZUSP*): **Brazil**, **Bahia**, Wenceslau Guimarães, Rio Patioba, 13°34’50.3”S, 39°42’17”W, 432m, 09.x.2010, pan light trap U.V./white lights (Calor, A.R., Quinteiro, F.B., e col.).

*Paratypes*: **Brazil**, **Bahia**, Wenceslau Guimarães, Rio Patioba, 13°34’50.3”S, 39°42’17”W, 432m, 09.x.2010, pan light trap U.V./white lights (Calor, A.R., Quinteiro, F.B., e col.)– 1 male (alcohol; UFBA); Wenceslau Guimarães, near the Station headquaters, 13°35’43.5”S, 39°43’11.9”W, 531m, v.2011, Malaise trap—2 males, 1 female (alcohol; UMSP); same data except, 10.x.2010 – 1 male (alcohol; UFBA); Uruçuca, Córrego Samambaia, 07.iv.2011, pan light trap, U.V./white lights—1 male, 1 female (alcohol; MZUSP); Ilhéus, around Parque Estadual da Serra do Conduru, 12.vii.2011, pan light trap U.V./white lights (Calor, A.R., Mariano, R., Quinteiro, F.B.)– 2 males (alcohol; UFBA).

*Distribution*: Brazil (BA).

*Etymology*: from Latin *clavos* = club, *cornus* = horn. The epithet is in reference to the club-shaped process over tergum X.

*Taxonomic and collection remarks*: this species seems to be morphologically related to *Oecetis peruviana* (Banks) [17], *O*. *scoparia* Flint [18] and *O*. *traini* Rueda-Martín, Gibon & Molina [6]. Tergum X and preanal appendages of all four species are similar, as are the inferior appendages, except for the small quadrate basal process in the new species. However, the phallic apparatus of each of these species is different; that of the new species is most similar to *Oecetis scoparia*. The clavate process with a ventral hump is an exclusive character of *Oecetis clavicornia* sp. nov. (*O*. *scoparia* has a clavate process, but the hump is dorsal). Additionally, *Oecetis peruviana*, *O*. *scoparia* and the new species share a setal brush on the base of the M vein.

This species was collected under the same conditions and in the same collecting sites as *Oecetis martinae* sp. nov. The only exception is for the specimens collected in Uruçuca, around the Conduru Mountains State Park, where the specimens were collected in shallow streams surrounded by a sparser rainforest than those in Wenceslau Guimarães. Like *Oecetis martinae* sp. nov., this species seems to be typical from higher altitudes. The immature stages and female of this species are unknown.

***Oecetis furcata* sp. nov.** urn:lsid:zoobank.org:act:F5C0B55C-BFBE-43BC-95C4-27042AE466B5

(Fig 4)

**Fig 4:**
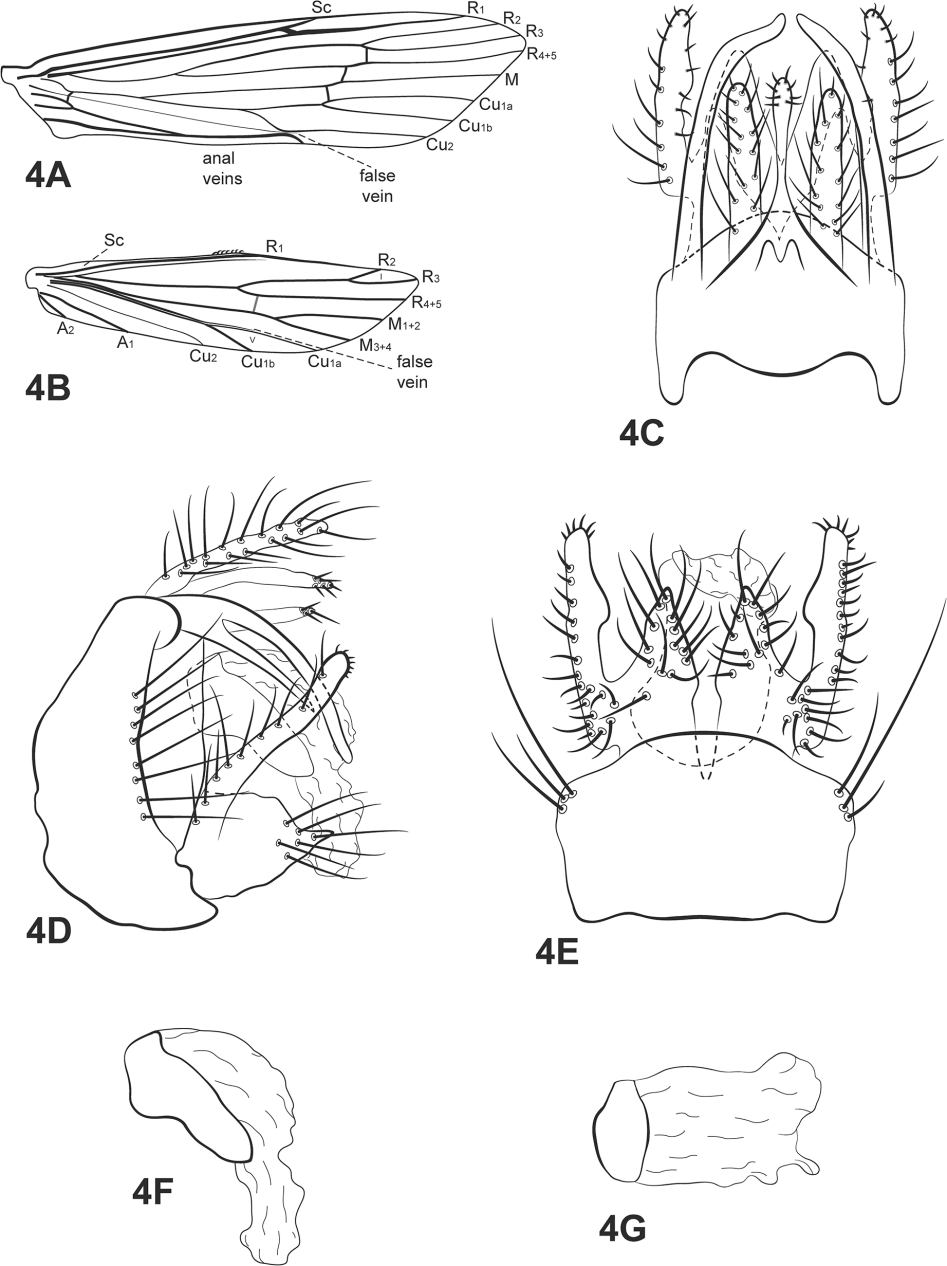
*Oecetis furcata* sp. nov. A, forewing. B, hind wing. C, male genitalia, dorsal view. D, male genitalia, lateral view. E, male genitalia, ventral view. F, phallic apparatus, lateral view. G, phallic apparatus, dorsal view. Abbreviation of the wing structures: Sc, subcosta; R, radius; M, medius; A, anal vein; I, fork 1; V, fork 5.

*Diagnosis*: This species is a member of the *Quaria* species group and can be diagnosed from its congeners by having dorsolateral processes on segment IX that are divided into ventral and dorsal lobes, with the ventral one shorter than the dorsal one. Also, the inferior appendages have a singular shape among species in the genus, with a long and terete dorsal lobe and a very acute, spine like distal lobe. The new species seems to be morphologically similar to *Oecetis falicia* Denning & Sykora [44], although the dorsal lobe on the dorsolateral process on *O*. *falicia* is very short and the distal lobe on the inferior appendage is not acute.

*Male*: body length 4.55 mm (n = 3). Forewing length 5.85 mm (n = 3).

Head: yellowish brown (alcohol). Antennae long, about 3 times length of forewings; scape and pedicel both short and stout, about same length as flagellomeres. Maxillary palps yellowish brown, 5-segmented, all segments subequal in length and width, densely covered with setae. Labial palps yellow, 3-segmented, first segment very small.

Thorax: pterothorax yellowish brown in dorsal region and pale yellow in lateral and ventral regions. Forewings yellowish brown with cord crossveins slightly thickened; wing vein pattern as Fig 4A and 4B; crossvein *m-cu* reaching Cu_1a_ (after branching of Cu into Cu_1a_ and Cu_1b_). Hind wings with basal brush; forks I and V present; false vein near Cu_1a_ (Fig 4B). Legs yellowish brown, mid and hind legs both with with row of small spines on tibia and tarsus. Tibial spur formula 1,2,2, fore tibial spur very small.

*Genitalia*: Segment IX annular and short; dorsolateral process present, long and bilobed, ventral lobe shorter than dorsal, with apex acute, dorsal lobe with apex rounded. Preanal appendage long and narrow, rod-like, with apex covered with setae (Fig 4C). Tergum X divided mesally by V-shaped incision for half of its length, forming two lobes, broad basally, tapering apically, apex acuminate; rod-like process above tergum X present, apex slightly enlarged, with few small setae (Fig 4C). Inferior appendage 1-segmented, broad basally; ventral lobe absent; dorsal lobe long, cylindrical, apex rounded, with setae along its length (Fig 4D), small hump present on mid inner portion (Fig 4E); distal lobe reduced, broad, with apex acute and covered with setae (Fig 4D and 4E). Phallic apparatus asymmetrical, broad basally, tapering apicad, spoon-shaped, bent ventrad (Fig 4F); endotheca longer than phallobase (Fig 4F and 4G).

*Holotype* (*male in alcohol; MZUSP*): **Brazil**, **Bahia**, Camacan, Reserva Particular do Patrimônio Natural Serra Bonita, stream 1, 31.iii.2011, pan light trap U.V./white lights (Quinteiro, F.B., França, D., Barreto, H.).

*Paratypes*: **Brazil**, **Bahia**, Camacan, Reserva Particular do Patrimônio Natural Serra Bonita, stream 1, 30.iii.2011, pan light trap U.V./white lights (Quinteiro, F.B., França, D., Barreto, H.)– 1 male (alcohol; UFBA); same data except Malaise trap 2, i.2009 – 1 male (alcohol; UMSP).

*Distribution*: Brazil (BA).

*Etymology*: from Latin *furcus* = fork. The epithet refers to the forked dorsolateral process on segment IX.

*Taxonomic and collection remarks*: based on the presence of the dorsolateral processes on segment IX, this new species is a member of *Quaria* species group. There are a few described species recognized in this group, such as *Oecetis falicia* Denning [44], *O*. *fibra* Chen & Morse *in* Quinteiro & Calor [7], *O*. *morsei* Bueno-Soria [45] and *O*. *rafaeli* Flint [24]. However, Chen included additional undescribed species in his unpublished thesis. Within *Quaria*, only *O*. *acanthostema* sp. nov. and *O*. *furcata* sp. nov. have the process over tergum X enlarged apically; all of the other members of the species group have the process rod-like. With regard to the shape of the phallic apparatus, the new species is similar to *O*. *falicia*, but it seems that the former has a shorter phallobase compared to the latter. This shape of phallic apparatus seems to be very common in *Quaria* group.

This species is known only from Serra Bonita, a reserve of Atlantic Forest in the south of Bahia State. It was collected near coldwater low order streams with dense surrounding rainforest. The immature stages and female of this species are unknown.

***Oecetis froehlichi* sp. nov.** urn:lsid:zoobank.org:act:571C3136-2333-4677-804C-B22A12DEE184

(Fig 5)

**Fig 5:**
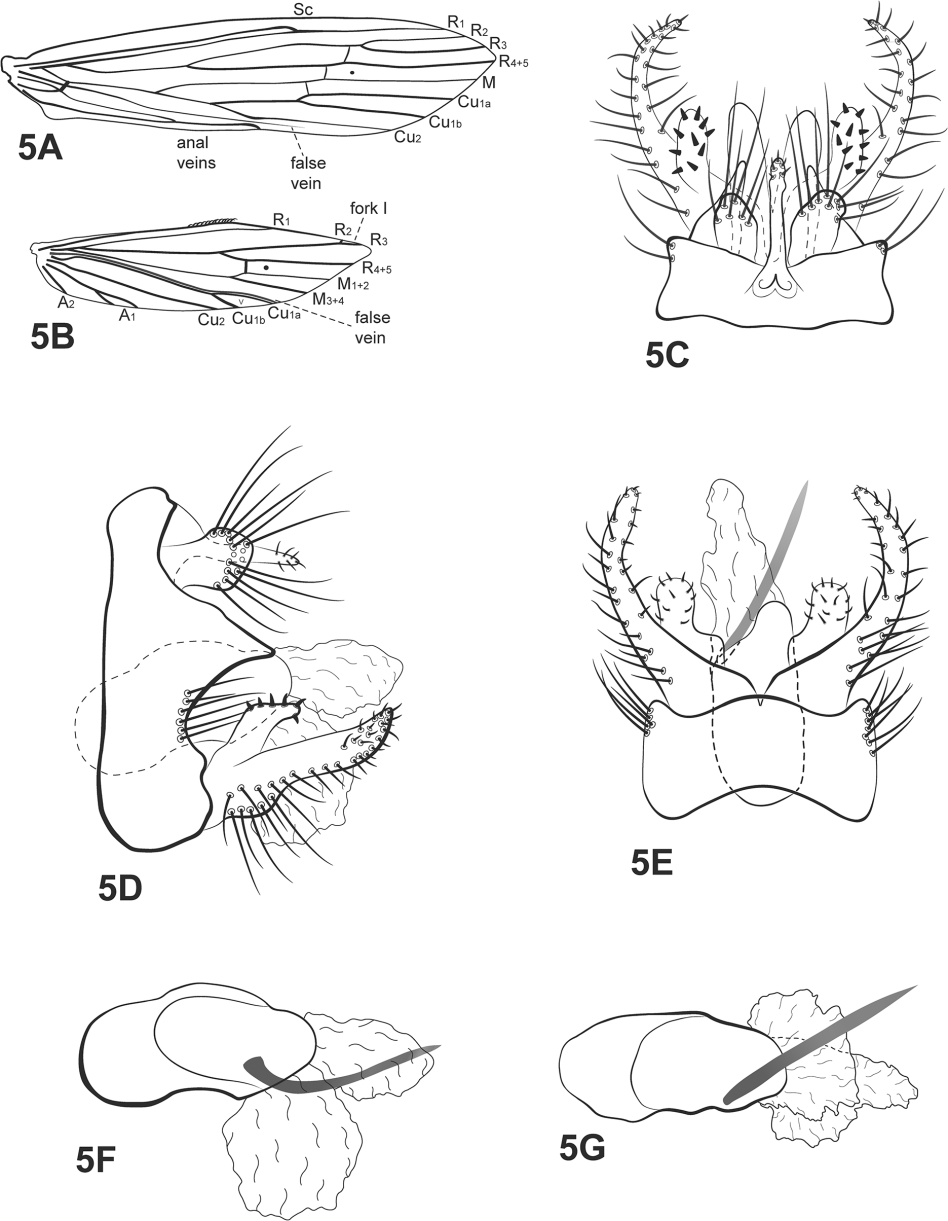
*Oecetis froehlichi* sp. nov. A, forewing. B, hind wing. C, male genitalia, dorsal view. D, male genitalia, lateral view. E, male genitalia, ventral view. F, phallic apparatus, lateral view. G, phallic apparatus, dorsal view. Abbreviation of the wing structures: Sc, subcosta; R, radius; M, medius; A, anal vein; I, fork 1; V, fork 5.

*Diagnosis*: this species is very distinctive, showing some singular features. The inferior appendages are similar to *O*. *scoparia* Flint [18], but *O*. *froehlichi* has a thumb-like dorsal lobe with spines over the surface, not found in any other species in the genus. Also, the inferior appendages are thinner than *O*. *scoparia*. The new species has an acuminate lateral projection on segment IX, as in *Oecetis osteni* Milne[16], but that of the new species is shorter. The new species also has a long phallic spine, as in Chen´s illustration of *O*. *excisa* [19] ([9] Fig 5 De, p. 587), but this spine is curved upward in *O*. *froehlichi*. The shape of the phallic apparatus is very different from *O*. *excisa*.

*Male*: body length 4.0 mm (n = 4). Forewing length 6.0 mm (n = 4).

Head: yellowish brown (alcohol). Antennae long, about 3 times length of forewings; scape and pedicel both short and stout. Maxillary palps yellowish brown, 5-segmented, all segments subequal in length and width, densely covered with setae. Labial palps yellow, 3-segmented.

Thorax: Pterothorax brown in dorsal region and pale yellow in lateral and ventral regions. Forewings hyaline, yellowish brown with dark bands over cord and dark spots over base of M, fork of Cu_1_ and Cu_2_ and at ends of M, Cu_1a_, Cu_1b_ and Cu_2_ (Fig 5A); wing vein pattern as Fig 5A and 5B; vein M-Cu reaching Cu_1a_ (after branching of Cu into Cu_1a_ and Cu_1b_). Hind wings with basal brush; forks I and V present; fork I very short; false vein near Cu_1a_ (Fig 5B). Legs yellow, mid legs each with row of small spines on tibia and tarsus. Tibial spur formula 0,2,2.

*Genitalia*: Segment IX annular and short; posterolateral projection of segment IX, expanded at base, acuminate (Fig 5C and 5D); mesodorsal process present, rod-like, apex covered with small setae (Fig 5D). Preanal appendage small, slightly longer than wide, ovoid, apex rounded and covered with setae (Fig 5C). Tergum X membranous, divided mesally by V-shaped incision on distal third, forming two lobes, broad basally, rounded apically (Fig 5C). Inferior appendage 1-segmented, curved medially, with small hump ventrally, tapering distally, acuminate at apex (Fig 5D); dorsal lobe present, thumb-like, somewhat widened apically, with apex rounded, covered with small thick setae (Fig 5D and 5E); ventral lobe absent; row of spine like setae on inner surface absent (Fig 5E). Phallic apparatus asymmetrical, slightly enlarged basally in dorsal view (Fig 5G); slightly bent ventrad, membranous apically; endotheca bilobate when everted (Fig 5F and 5G); phallic spine present, long, curved upward in lateral view (Fig 5F).

*Holotype* (*male in alcohol; MZUSP*): **Brazil**, **Bahia**, Wenceslau Guimarães, Estação Ecológica Estadual Wenceslau Guimarães, Riacho Serra Grande, pan light trap U.V./white lights, 10.x.2010 (Calor, A.R., Quinteiro, F.B. & col.).

*Paratypes*: **Brazil**, **Bahia**, Wenceslau Guimarães, Estação Ecológica Estadual Wenceslau Guimarães, Riacho Serra Grande, pan light trap U.V./white lights, 10.x.2010 (Calor, A.R., Quinteiro, F.B. & col.)– 2 males (alcohol; UFBA); 13°35’38”S, 39°42’50”W, 08.x.2010, light trap U.V./white lights, (Calor, A.R., Quinteiro, F.B. & col.)– 1 male (pinned; UMSP).

*Distribution*: Brazil (BA).

*Etymology*: this species epithet honors Prof. Dr. Claudio G. Froehlich (Universidade de São Paulo), a great Brazilian entomologist, who has been instrumental in contributing to our knowledge of Neotropical aquatic insects.

*Taxonomic and collection remarks*: this new species shares a few characters with some congeners. Its tergum X is similar to *O*. *scoparia*, *O*. *traini* and *O*. *clavicornia* sp. nov., as is the shape of the inferior appendage. However, the shape of the phallic apparatus and phallic spine are unique. In addition, the bilobate endotheca is not common in *Oecetis*. This more complex endotheca seems to be present more often in members of *Quaria* species group. However, the posterolateral processes in *O*. *froehlichi* does not seem to be homologous to those dorsolateral processes that define the *Quaria* group. In this way, it seems that this species could not be in the *Quaria* group. Even so, the presence of a phallic spine, and complex endotheca may indicate an affinity with the *Quaria* species group. Additionally, an m-shaped mark can be seen after the clearing process on the base of the mesodorsal process above tergum X. This mark was not found in any other species of *Oecetis*.

This species is known only from Estação Ecológica Wenceslau Guimarães and the adults were collected with light traps. The environmental conditions were the same as for *Oecetis martinae* sp. nov., *Oecetis clavicornia* sp. nov. and *Oecetis furcata* sp. nov. The immature stages and female of this species are unknown.

### Additional species records and synopsis of Brazillian species

In an attempt of facilitate *Oecetis* identification, and to provide a foundation for subsequent works on morphology, we present redescriptions and new illustrations of species of *Oecetis* previously recorded from Brazil, including new characters and a standardized terminology. We also provide new distribution records. The primary distributions were based on Flint *et al*. [46], Blahnik *et al*. [47], Paprocki *et al*. [25], Dumas *et al*. [26], Rueda-Martín *et al*. [6], Santos *et al*. [48], Quinteiro *et al*. [28] and Henriques-Oliveira *et al*. [8]. The new records are highlighted in bold. The examined material is deposited in UFBA.

*Oecetis amazonica* (Banks, 1924)

(Fig 6)

**Fig 6:**
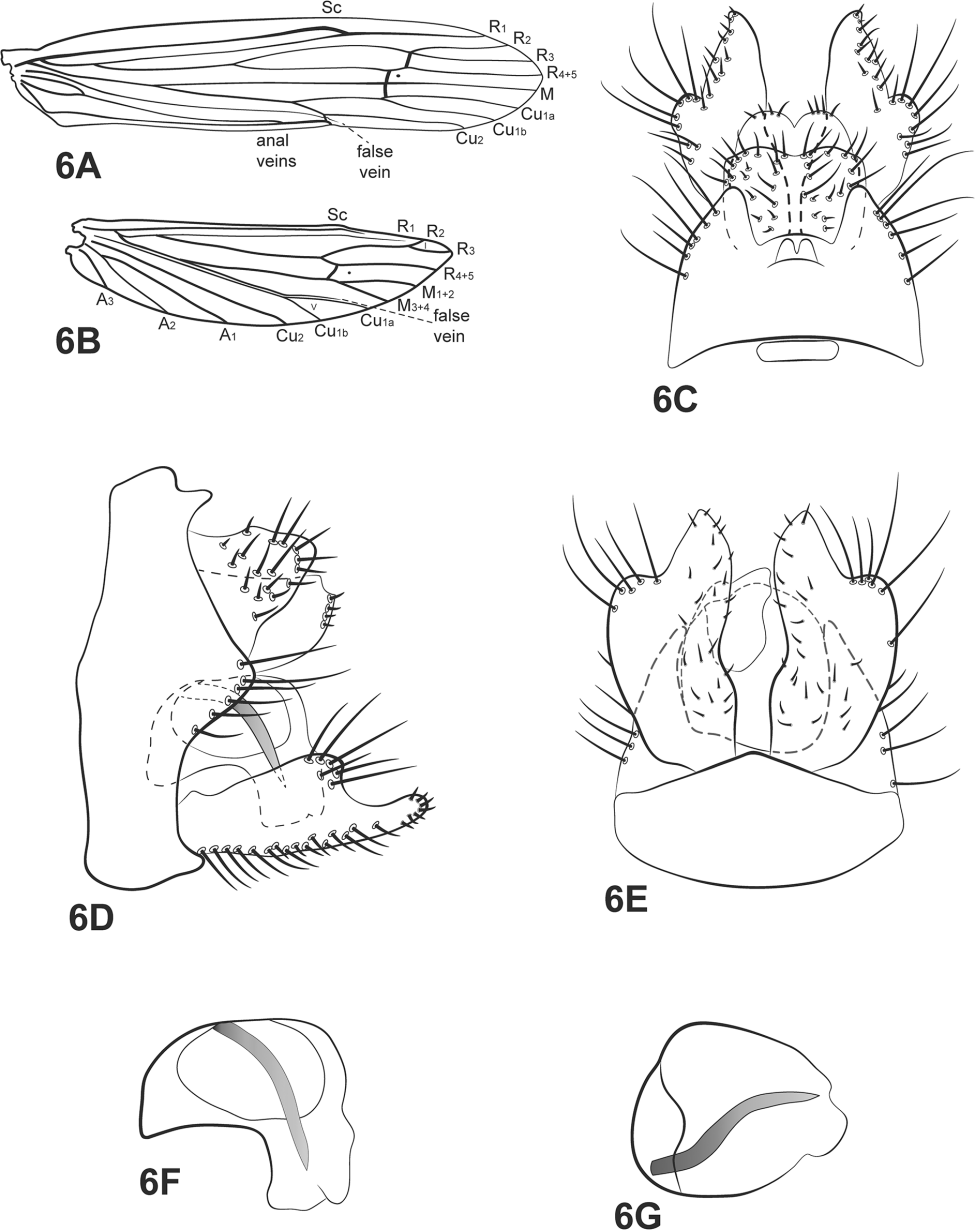
*Oecetis amazonica* Banks. A, forewing. B, posterior wing. C, male genitalia, dorsal view. D, male genitalia, lateral view. E, male genitalia, ventral view. F, phallic apparatus, lateral view. G, phallic apparatus, dorsal view. Abbreviation of the wing structures: Sc, subcosta; R, radius; M, medius; A, anal vein; I, fork 1; V, fork 5.

*Oecetina amazonica* Banks, 1924 [17]: 447

*Male*: Forewing length 8–10 mm [23].

Head: color yellowish brown [17]. Antennae long, about 3 times length of forewings; scape and pedicel both short and stout, about same length as segments of flagellum. Maxillary palps brown, 5-segmented, all segments subequal in length and width, densely covered with setae. Labial palps yellow, 3-segmented, first segment very small, covered with brown setae.

Thorax: pterothorax yellowish brown [17]. Forewings brown [17,23]; dark bands over *s*, *r-m* and *m-cu*; area around cord hyalinized; dark spots absent; *m-cu* crossvein reaching fork V (Fig 6A). Hind wings with forks I and V [17]; basal brush present (Fig 6B). Legs yellowish brown; mid legs each with row of spines on distal half of femur and on tibia and tarsus; hind legs each with row of spines on tarsus. Tibial spur formula 0,2,2.

*Genitalia*: Segment IX annular and short; acrotergite present mesodorsally. Preanal appendages fused to each other, short and rounded [9], setose (Fig 6D). Rod-like process above tergum X absent. Tergum X membranous, with a shallow V-shaped incision apically, dorsal view, composed of a single elongate lobe, broad basally, with a shallow depression distad (Fig 6C). Inferior appendages broad basally [9,23], covered with setae; dorsal lobe broad and rounded; ventral lobe absent; distal portion broad [23], acuminate, forming with dorsal lobe an L-shaped incision (Fig 6D); small spine-like setae absent (Fig 6E). Phallic apparatus curved downward, short [9] (Fig 6F); rounded and inflated, in dorsal view; phallic spine present, curved downward [9].

*Material examined*: **Brazil**, **Bahia**, Curaçá, Recanto Campestre, Rio São Francisco, 08°59’57.6”S, 39°54’47.2”W, 06.v.2011 (Silva-Neto, A.M.)– 3 males, 4 females (alcohol); same data except light trap (França, D.)– 4 males (pinned); Pilão Arcado, Barra do Brejo, 03.iv.2008, light trap U.V./white lights (Alvim, Silva-Neto, Rebouças, Rezende)– 1 male (alcohol); **São Paulo**, Populina, Rio Grande, Porto Amaral, 19°20’49”S, 50°31’25.23”W, 335m, 12.vii.2010, light trap U.V./white lights (Calor, A.R.)– 3 males, 2 females (alcohol).

*Distribution*: Argentina, Peru, Venezuela, Bolivia, Brazil [AM, **BA (new record)**, **SP (new record)**].

*Taxonomic remarks*: the only divergence between the original description [17] and the characteristics added by Chen [9] and Flint [23] is the body color. Banks [17] pointed out that the specimens have a yellowish brown head and body. The examined specimens are a distinctly dark brown color for both characters.

*Oecetis connata* Flint, 1974

(Fig 7)

**Fig 7:**
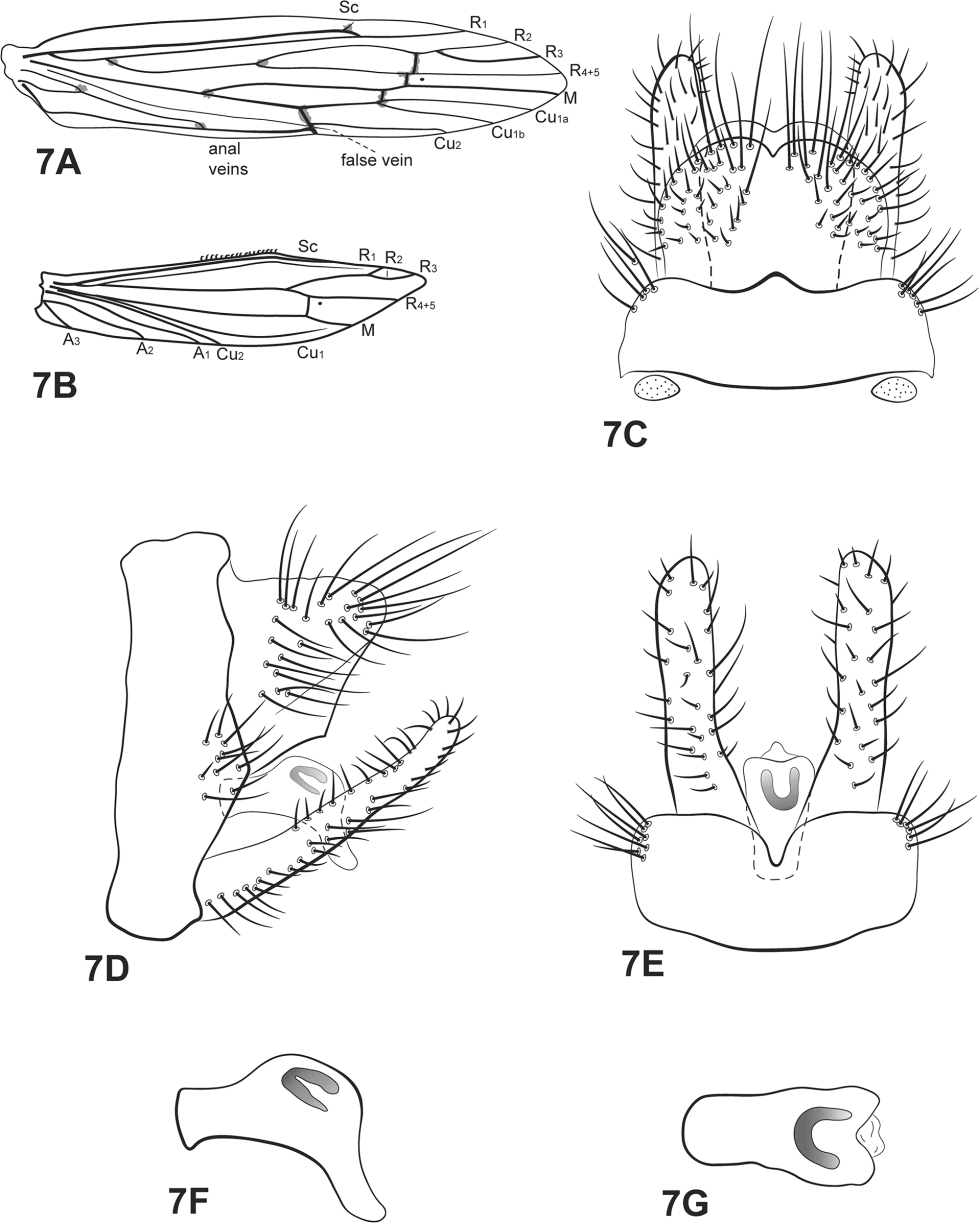
*Oecetis connata* Flint. A, forewing. B, hind wing. C, male genitalia, dorsal view. D, male genitalia, lateral view. E, male genitalia, ventral view. F, phallic apparatus, lateral view. G, phallic apparatus, dorsal view. Abbreviation of the wing structures: Sc, subcosta; R, radius; M, medius; A, anal vein; I, fork 1; V, fork 5.

*Oecetis connata* Flint, 1974 [18]: 122

*Male*: Forewing length 6.5 mm [18].

Head: color yellowish brown [18]. Antennae long, about 3 times length of forewing; scape and pedicel both short and stout, about same length as segments of flagellum. Maxillary palps yellow, 5-segmented, all segments subequal in length and width, covered with brown setae. Labial palps yellow, 3-segmented, first segment very small, covered with brown setae.

Thorax: pterothorax yellowish brown dorsally [18], pale yellow in lateral and ventral views. Forewings yellowish brown [18]; dark bands over *s*, *r-m* and *m-cu* [18]; dark spots on forks, junctions and end of veins [18]; crossvein *m-cu* reaching fork V or Cu_1a_ (after branching into Cu_1a_ and Cu _1b_) (Fig 7A). Hind wings with fork I [18]; basal brush present (Fig 7B). Legs yellowish brown; mid legs each with row of spines on femur, tibia and tarsus; hind legs each with row of spines on tarsus. Tibial spur formula 1,2,2, fore tibial spur very small.

*Genitalia*: Segment IX annular and short [18]; two small acrotergites present dorsolaterally. Preanal appendages fused with tergum X, forming a hoodlike structure [18], dorsal view (Fig 7C). Rod-like process above tergum X absent. Inferior appendages slightly enlarged basally [18]; dorsal lobe absent; ventral lobe absent; distal portion narrow [18], tapering posteriorly with apex acuminate (Fig 7D); small spine-like setae absent (Fig 7E). Phallic apparatus curved downward, short, comma shaped, with elongate ventral tip [18] (Fig 7F); phallic spine absent [9]; phallotremal sclerite horseshoe shaped (Fig 7G).

*Material examined*: **Brazil**, **Bahia**, Andaraí, Igatu, Rio Coisa Boa, 12°53’27.7”S, 41°19’0.0”W, 673m, 12.iii.2011, pan light trap 2, U.V./white lights (Calor, A.R., Camelier, P.)– 5 males, 7 females (alcohol); 12°53’33.7”S, 41°18’58.3”W, 664m, light trap U.V./white lights, 11.iii.2011 (Calor, A.R., Camelier, P., Zanata, A.)– 1 male, 3 females (alcohol); Mucugê, Parque Sempre Vivas, Rio Piabinha, 12°59’34”S, 41°20’27”W, 921m, 25.vii.2010, pan light trap U.V./white lights (Calor *et al*.)– 1 male (alcohol); Parque Nacional da Chapada Diamantina, Cachoeira da Garapa, 17°44’80.7”S, 41°20’87.7”W, 340m, 25.x.2008, light trap U.V./white lights (Calor, A.R., Mariano, R., Mateus, S.)– 2 males (pinned); Barreiras, rio de Janeiro, Cachoeira Acaba Vidas, 11°53’67.3”S, 45°36’09.6”W, 722m, 14.x.2008, light trap U.V./white lights (Calor, A.R., Mariano, R., Mateus, S.)– 3 males (pinned); Nova Redenção, Fazenda Moreno, Rio Paraguaçu, 12°46’44”S, 41°09’08”W, 26.vii.2010, light trap U.V./white lights (Calor, A.R. *et al*.)– 1 male, 6 females (alcohol); **Mato Grosso**, Nova Xavantina, Córrego Benedito Ferreira, 06.xii.2006, light trap U.V./white lights (Calor, A.R., Mariano, R.M.L., Mateus, S.)– 6 males, 10 females (alcohol); Fazenda Buriti, 06.i.2000, light trap U.V./white (Mendes, H.F.)– 1 male (alcohol); Ribeirão Cascalheira, Posto de Saúde road, 2^nd^ bridge, 26.xi.2006, pan light trap U.V./white lights (Calor, A.R., Silva, R.M, Mateus, S.)– 3 males, 6 females (alcohol); Fazenda Campina Verde, Rio Suiamissu, 28.xi.2006, pan light trap U.V./white lights (Calor, A.R., Silva, R.M., Mateus, S.)– 6 males, 7 females (alcohol); Fazenda Canguru road, stream of the dam, 27.xi.2006, pan light trap U.V./white lights (Calor, A.R., Silva, R.M., Mateus, S.)– 9 males, 26 females (alcohol).

*Distribution*: Guyana, Surinam, Brazil [AM, BA, **MT (new record)**, PA].

*Taxonomic remarks*: according to Chen [9], *Oecetis connata*, *O*. *punctipennis* and *O*. *iguazu* form a small group within *Oecetis* with the comma shaped phallic apparatus as a grouping character. *Oecetis dominguezi* can also be included in this group due to its shared comma shaped phallic apparatus. *Oecetis connata* is easily separated from the other three species by its preanal appendages fused to one another, and also fused to tergum X, forming, according to Flint [18], a single, hood-like structure. No other described species in the Neotropics has this characteristic. Also, *O*. *connata* is the only species in the Neotropical region that does not have fork V in the hind wings (Fig 7B). The position of the *m-cu* crossvein was variable in the examined material.

*Oecetis excisa* Ulmer, 1907

(Figs 8; 9A)

**Fig 8:**
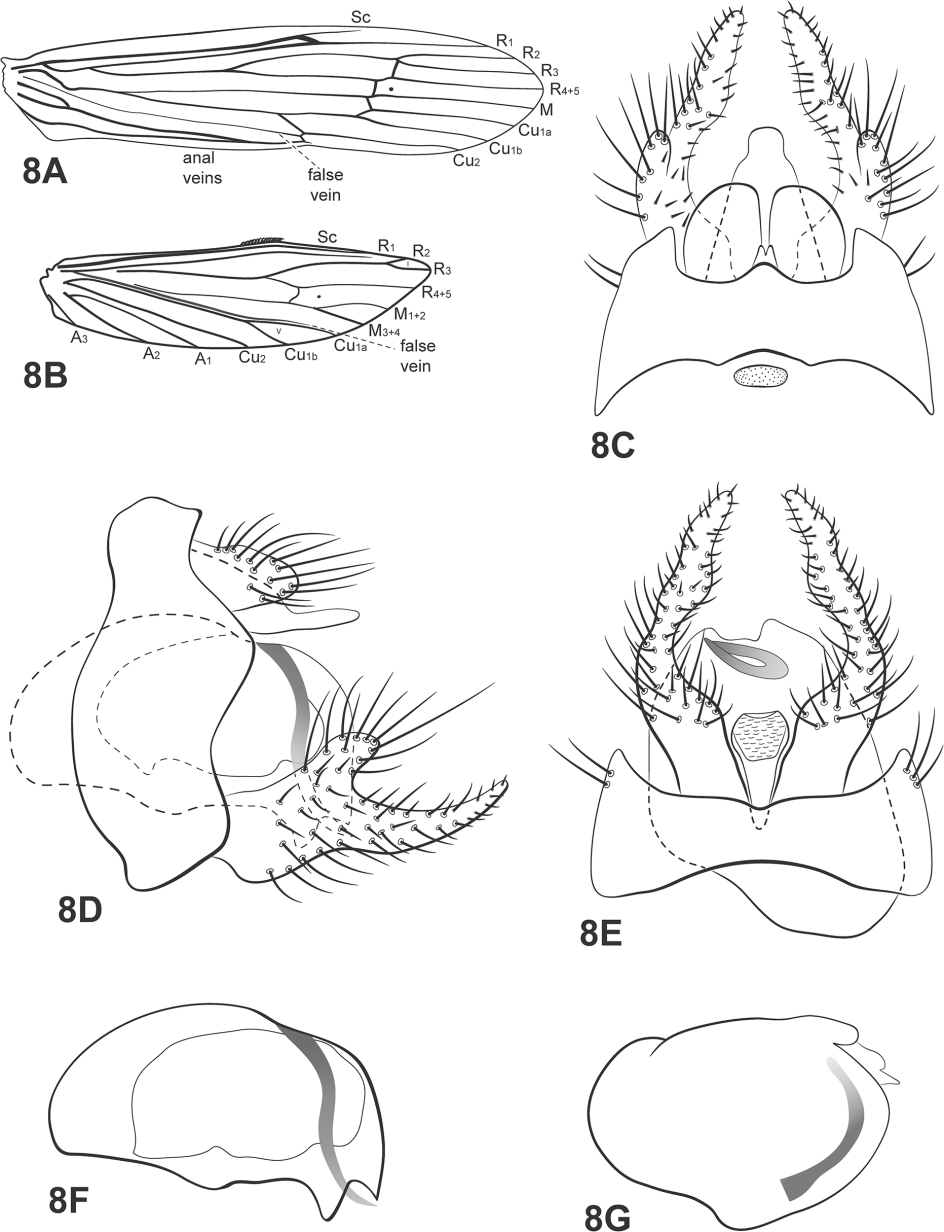
*Oecetis excisa* Ulmer. A, forewing. B, hind wing. C, male genitalia, dorsal view. D, male genitalia, lateral view. E, male genitalia, ventral view. F, phallic apparatus, lateral view. G, phallic apparatus, dorsal view. Abbreviation of the wing structures: Sc, subcosta; R, radius; M, medius; A, anal vein; I, fork 1; V, fork 5.

**Fig 9:**
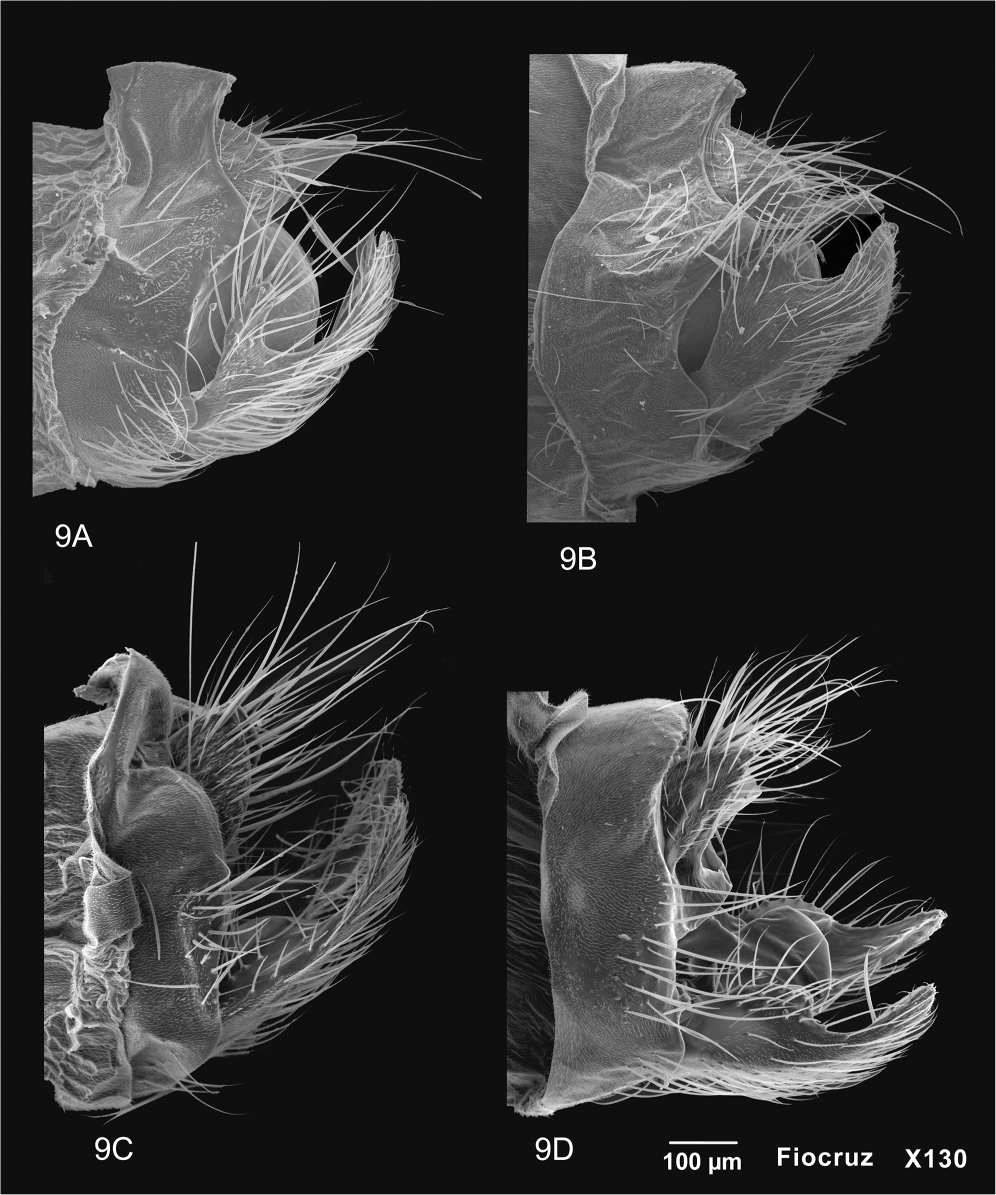
Scanning Eletron Microscope of four *Oecetis* species. A, *Oecetis excisa* Ulmer, male genitalia, lateral view. B, *Oecetis inconspicua* (Walker), male genitalia, lateral view. C, *Oecetis iguazu* Flint, male genitalia, lateral view. D, *Oecetis punctipennis* (Ulmer), male genitalia, lateral view.

*Oecetis excisa* Ulmer 1907: 15[19].

*Oecetis mutila* Navás 1918: 22 (male) [49]; Schmid 1949: 382 [50] (to synonymy).

*Oecetis castilleja* Navás 1920: 134 [51] (female); Schmid 1949: 382 [50] (possible synonym of *O*. *excisa*); Flint 1972: 244 [52] (to synonymy).

*Oecetis muhnia* Navás 1920b: 28 [53] (male); Flint 1972: 244 [52] (to synonymy).

*Oecetis apicata* Navás 1931: 323 [54] (female); Flint 1982b: 50 [23] (to synonymy).

*Male*: body length 5–6 mm. Forewing length 8 mm [19].

Head: color yellowish brown. Antennae long, about 2.5 times length of forewings; scape and pedicel both short and stout, about same length as the segments of flagellum. Maxillary palps brown, covered with setae, 5-segmented, segments subequal [19]. Labial palps yellow, 3-segmented, first segment very small.

Thorax: pterothorax yellowish-brown [19], dorsal view. Forewings yellowish brown, hyaline [19]; dark bands over *s*, *r*-*m* and *m*-*cu* [23]; dark spots absent; *m*-*cu* crossvein reaching fork V (Fig 8A). Hind wings with forks I and V [6,19] (Fig 8B). Legs light yellow [19]; tibial spur formula 1,2,2 [19].

*Genitalia*: Segment IX annular and short; one small acrotergite present mesodorsally. Preanal appendages short and rounded, setose. Rod-like process above tergum X absent. Tergum X membranous, undivided in dorsal view [6]; composed of a single elongate lobe, broad basally, digitate apically, abruptly narrowed in dorsal view (Fig 8C). Inferior appendages broad basally, covered with setae [19]; dorsal lobe acute, narrow basally; ventral lobe absent; distal portion narrow, tapering posteriorly, with apex acuminate; forming with dorsal lobe a deep C-shaped incision (Fig 8D); small spine-like setae absent (Fig 8E). Phallic apparatus curved downward, large, rounded and inflated, extending the length of segment IX (Fig 8F); phallic spine present, curved helically, counter clockwise (Fig 8G).

*Material examined*: **Brazil**, **Bahia**, Curaçá, Rio Buracão, 09°08’02.1”S, 39°58’43.6”W, 362m, 06.v.2011, pan light trap U.V./white lights (França, D.)– 1 male, 5 females (alcohol); River São Francisco, Pousada Recanto Campestre, 08°59’58.7”S, 39°54’48.3”W, 415m, 03.v.2011, pan light trap U.V./white lights, (França, D.)– 1 male, 2 females (alcohol); same data except 06.v.2011, light trap U.V./white lights (França, D.)– 1 male (pinned); Rancho do Tio Zé, tributary of Rio Buracão, 09°07’48.1”S, 39°58’45.7”W, 362m, 05.v.2011, pan light trap U.V./white lights (França, D.)– 1 male (alcohol); Rio Barra Grande, under the bridge, 09°06’53.5”S, 39°56’13.4”W, 415m, 05.v.2011, light trap U.V./white lights (França, D.)– 1 male, 3 females (alcohol), 1 male (pinned); same data except 04.v.2011, light trap U.V./white lights (França, D.)– 1 male (pinned/SEM); 08°59.960’S, 39°54.787’W, 347m, 06.v.2011, light trap U.V./white lights (Silva-Neto, A.M)– 2 males, 3 females (alcohol); Iaçú, Rio Paraguaçú, 12°41’11”S, 40°07’08”W, 15.v.2010, light trap U.V./white lights—1 male (alcohol); same data except (França, D., Burger, R.)– 1 male (pinned/ SEM); Mucugê, Parque Nacional da Chapada Diamantina, Rio Piabinha, 25.vii.2010 – 1 male (alcohol); Igatu, Rio Coisa Boa, 12°53’33”S, 41°19’0”W, 12.v.2010, light trap U.V./white lights (França, D., Burger, R.)– 1 male (pinned); Pindobaçú, Cachoeira da Fumaça, 16.xii.2009, 10º28’43”S, 40°12’17.6”W (Zacca, T)– 1 male (alcohol); **Ceará**, Crato, Sítio Fundão, Rio Batateiras, 07°13’47.7”S, 39°26’08.4”W, 436m, 07.ii.2011, light trap U.V./branca (Quinteiro, F.B., Costa, A.M.)– 1 male, 2 females (alcohol), 1 male (pinned); **Mato Grosso**, Ribeirão Cascalheira, Fazenda Campina Verde, Rio Suiamissú, light trap U.V./white lights, 28.xi.2006 (Calor, A.R., Mariano, R., Mateus, S.)– 1 male; **Paraíba**, Barra de Santana, Rio Paraíba, bridge BR-104, 07°31’44.3”S, 35°59’55”W, 336m, 31.vii.2009, light trap U.V./white lights (Calor, A.R., Lecci, L.S.)– 5 males, 5 females (pinned); **Rio Grande do Norte**, Serra Negra, Estação Ecológica do Seridó, Represa dos Campos, 06°34’50.8”S, 37°15’20”W, 205m, 27.vii.2009, light trap U.V./white lights (Calor, A.R., Lecci, L.S.)– 5 males, 3 females (alcohol); Caicó, Rio Sabugi, bridge, 06°26’52.7”S, 37°08’23.8”W, 141m, 27.vii.2009, light trap U.V./white lights (Calor, A.R., Lecci, L.S.)– 5 males, 17 females (pinned).

*Distribution*: Argentina, Bolivia, Mexico, Paraguay, Venezuela, Brazil [BA, CE, **MT (new record)**, **PB (new record)**, **RN (new record)**].

*Taxonomic remarks*: Ulmer’s description matches the specimens examined, except for one character: the tibial spur formula on the specimens observed was 0,2,2. No fore tibial spur was present, even in small specimens. *Oecetis excisa* and *O*. *inconspicua* are easily recognized by their large, rounded and inflated phallic apparatus bearing a curved phallic spine. However, the distinction between them is difficult because of the morphological similarities between their genitalia. Flint [23] and Rueda-Martín *et al*. [6] stated that it is possible that these species may be synonymous. However, in comparing identified material from UMSP (see material examined section) it was possible to find some small differences between the two species. They have a similar tergum X, preanal appendages and phallic apparatus, but the inferior appendages are different.

*Oecetis excisa* has a narrower dorsal lobe than *O*. *inconspicua*. Also, the apex of the inferior appendage seems to be longer in *O*. *excisa*, with the result that, in ventral view, it appears to project further beyond the C-shaped basomesal invagination of the appendage. These character differences were easily recognizable when both species were compared using Scanning Electron Microscopy (Fig 9A and 9B). The morphological differences pointed out by Rueda-Martín *et al*. [6] concerning the shape of tergum X (digitate in *O*. *excisa* and trapezoidal in *O*. *inconspicua*) and hind wing venation (*O*. *excisa* with fork I and *O*. *inconspicua* without) were not observed. Both species had fork I and the specimens examined had digitate as well as trapezoidal tergum X.

*Oecetis iguazu* Flint, 1983

(Fig 10; 9C)

**Fig 10:**
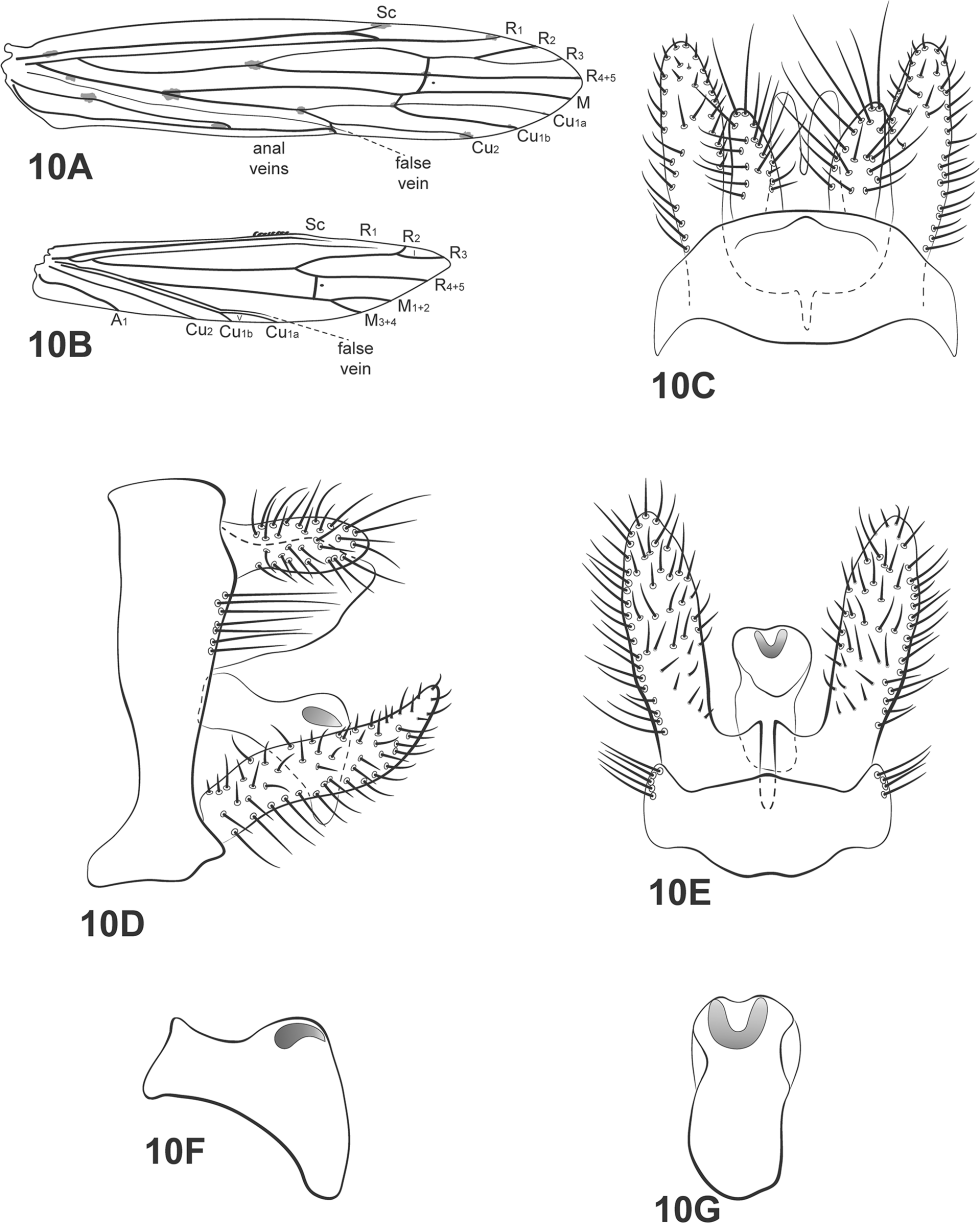
*Oecetis iguazu* Flint. A, forewing. B, hind wing. C, male genitalia, dorsal view. D, male genitalia, lateral view. E, male genitalia, ventral view. F, phallic apparatus, lateral view. G, phallic apparatus, dorsal view. Abbreviation of the wing structures: Sc, subcosta; R, radius; M, medius; A, anal vein; I, fork 1; V, fork 5.

*Oecetis iguazu* Flint, 1983 [20]: 70.

*Male*: body length 5.2 mm (n = 5). Forewing length 8 mm [20].

Head: color yellow [20]. Antennae long, about 3 times length of forewing; scape and pedicel both short and stout, about same length and width as the flagellar segments. Maxillary palps yellowish brown, 5-segmented, all segments subequal in length and width, densely covered with setae. Labial palps yellow, 3-segmented, first segment very small.

Thorax: pterothorax yellowish brown dorsally [20]; yellow in lateral and ventral views. Forewings yellowish brown, hyaline [20]; dark bands over cord absent; dark spots on forks, junctions and end of veins [20]; *m-cu* crossvein reaching Cu or fork V (Fig 10A). Hind wings with forks I and V [20]; basal brush present (Fig 10B). Legs yellow; mid legs each with row of spines on tibia and tarsus; hind legs each with row of spines on tarsus. Tibial spur formula 1,2,2, fore tibial spur very small.

*Genitalia*: Segment IX annular and short [20]; acrotergite absent. Preanal appendages slightly longer than wide (ovoid) [20], with setae. Rod-like process above tergum X absent. Tergum X membranous, divided mesally, with V-shape incision, broad basad and acute apicad in dorsal view (Fig 10C). Inferior appendage not enlarged basally, setose; dorsal lobe absent; ventral lobe absent; distal portion broad [20], apex acuminate (Fig 10D); small spine-like setae absent (Fig 10E). Phallic apparatus, in lateral view, curved downward, short, comma shaped, with ventral elongated tip, constricted in middle line [20] (Fig 10F); phallic spine absent [9]; phallotremal sclerite horseshoe shaped (Fig 10G).

*Material examined*: **Brazil**, **Bahia**, Lençóis, Parque Nacional da Chapada Diamantina, Rio Santo Antônio, 12°29.579’S, 41°19.752”W, 340m, 26.x.2008, light trap U.V./white lights (Calor, A.R., Mariano, R., Mateus, S.)– 2 males, 1 female (alcohol), 1 male (pinned); Abaíra, Rio Toborô, 13°17’35”S, 41°44’47”W, 28.vii.2010, light trap U.V./white lights (Calor, A.R. *et al*.)– 1 male (alcohol), 4 males (pinned); Abaíra-Piatã, Ouro Verde old road, Rio Toborô, 28.vii.2010, pan light trap U.V./white lights (Calor, A.R. *et al*.)– 3 males, 1 female (alcohol); Andaraí, Cachoeira da Garapa, 17°44’80.7”S, 41°20’87.7”W, 340m, 25.x.2008, light trap U.V./white lights (Calor, A.R., Mariano, R., Mateus, S.)– 1 male (pinned), 1 male (pinned; SEM); Mucugê, Parque Sempre Vivas, Rio Piabinha, 12°59’34”S, 41°20’29”W, 14.v.2010, light trap U.V./white lights (França, D., Burger, R.)– 3 males (pinned), 1 male (pinned/ SEM); Iaçu, Fazenda Touros, Rio Paraguaçú, 12°41’11”S, 40°07’08”W, 15.v.2010, light trap U.V./white lights (França, D., Burger, R.)– 1 male, 4 females (pinned).

*Distribution*: Argentina, Paraguay, Brazil [BA, ES, MG, SC, SP].

*Taxonomic remarks*: comparing the examined specimens with the description provided by Flint [20], the general color of the Bahia specimens was darker than that reported by Flint. The head is yellowish brown and the pterothorax is almost brown, in dorsal view.

As mentioned above, *Oecetis iguazu*, *O*. *connata* and *O*. *punctipennis* form a small group within *Oecetis* sharing the comma shaped phallic apparatus as one of the most conspicuous diagnostic characters [9]. Because the differences between *Oecetis iguazu* and *Oecetis punctipennis* are very subtle, the diagnosis of these two species is frequently difficult.

Both of them have identical preanal appendages, in dorsal and lateral views. Tergum X is also identical in dorsal view. However, in lateral view, *O*. *iguazu* has the apex of tergum X slightly acute, while *O*. *punctipennis* has a rounded one. However, these differences are seen only when both species are compared side-by-side. The phallic apparatus is comma shaped and, as already mentioned, identical in these three species.

Thus, the best character to distinguish *Oecetis iguazu* and *Oecetis punctipennis* is the shape of the inferior appendages, in lateral view (Figs 10D and 13D). *Oecetis iguazu* has the inferior appendages without lobes or with a smooth hump dorsally. In *O*. *punctipennis*, a pronounced dorsal expansion on the inferior appendages is noticeable and the apex is more acute than *O*. *iguazu*. These differences are clearly seen with a Scanning Electron Microscope (Fig 9C and 9D).

*Oecetis inconspicua* (Walker, 1852)

(Figs 11 and 9B)

**Fig 11:**
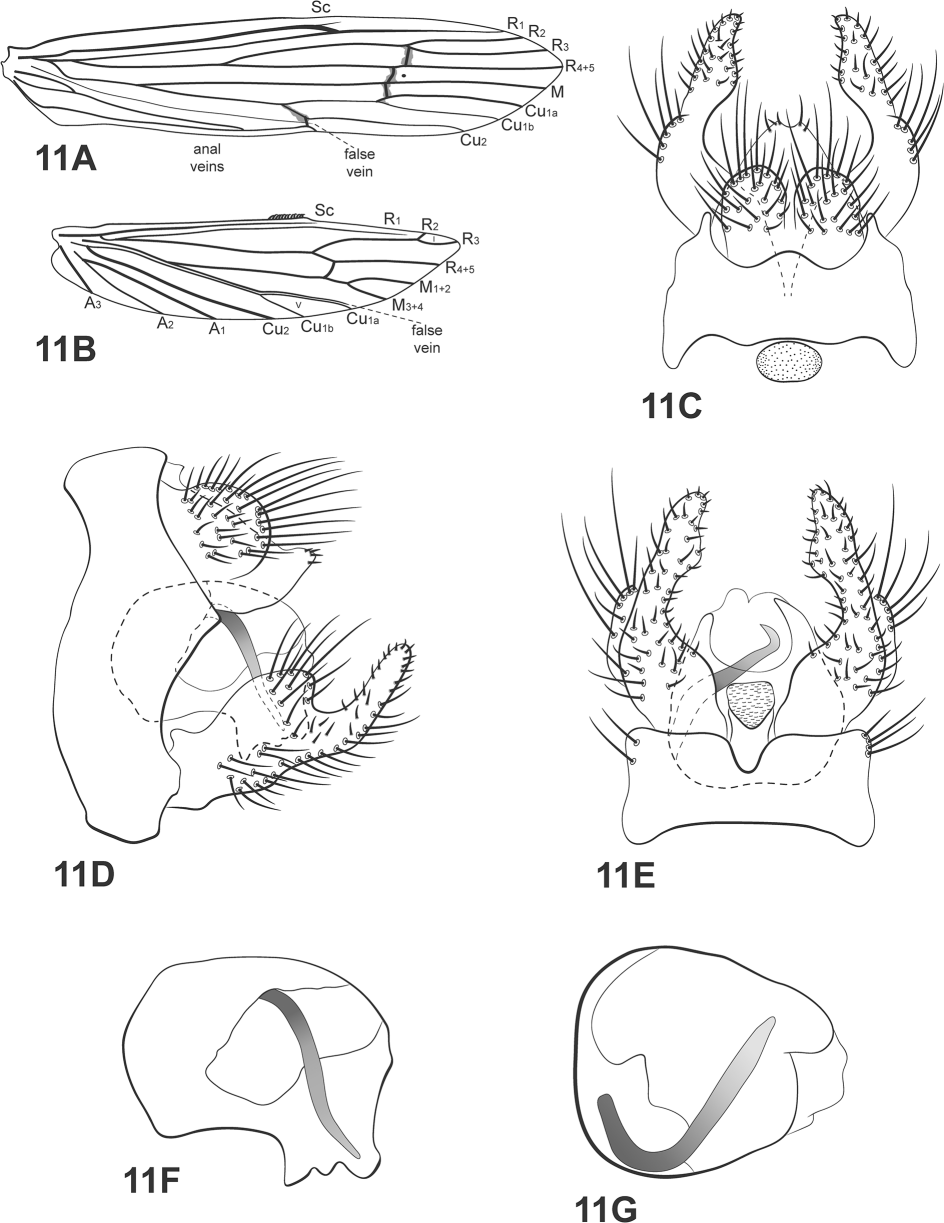
*Oecetis inconspicua* (Walker). A, forewing. B, hind wing. C, male genitalia, dorsal view. D, male genitalia, lateral view. E, male genitalia, ventral view. F, phallic apparatus, lateral view. G, phallic apparatus, dorsal view. Abbreviation of the wing structures: Sc, subcosta; R, radius; M, medius; A, anal vein; I, fork 1; V, fork 5.

*Leptocerus inconspicuus* Walker, 1852 [21]: 71–72

*Setodes flaveolata* Hagen, 1861: 282 [55] (male, female); Ross 1938: 24 [56] (synonymy; determined by Milne).

*Setodes micans* Hagen, 1861: 283 [55] (male, female); Ross, 1938: 25 [56] (synonymy; determined by Milne).

*Setodes sagitta* Hagen, 1861: 284 [55] (female); Banks, 1907: 46 [57] (in *Oecetina*); Ross, 1938: 25 [56] (synonymy; determined by Milne).

*Oecetina parvula* Banks, 1899:215 [58] (female); Ross, 1938: 25 [56] (synonymy; determined by Milne).

*Leptocerus flaveolatus* Banks, 1899: 214 [58] (in *Oecetina*); Betten, 1934: 269 [59] (to *Oecetis*).

*Oecetina flavida* Banks, 1899: 216 [58]; Ross, 1938: 24 [56] (synonymy; determined by Milne). *Oecetina inornata* Banks, 1907: 128 [60]; Milne, 1934: 19 [16] (to synonymy).

*Oecetina apicalis* Banks, 1907: 129 [60] (male); Milne, 1934: 19 [16] (to synonymy); Betten, 1934: 274.

*Oecetina antillana* Banks, 1938: 298 [61] (male); Flint, 1967: 23 [62] (lectotype, to synonymy).

*Male*: body length 5.29 mm (2.5 lines in Walker [21]). Forewing length 7–7.5 mm [9].

Head: color brown [10]. Antennae long, 3 times length of body [21]; scape and pedicel both short and stout, about same length as flagellar segments. Maxillary palps brown, covered with setae [21]; 5-segmented, all segments subequal in length and width. Labial palps yellow, 3-segmented, first segment very small, covered with brown setae.

Thorax: pterothorax brown in dorsal view [21], yellowish brown in lateral and ventral views. Forewings brown [21], dark bands over *s*, *r*-*m* and *m*-*cu* [21]; dark spots absent (Fig 11A); *m-cu* crossvein reaching different positions on Cu vein [21]. Hind wings with forks I and V [38], although Rueda-Martín *et al*. [6] say that fork I is absent); basal brush present (Fig 11B). Legs brown (testaceous according to Walker [21]); mid legs each with row of spines on distal half of femur, tibia and tarsus; hind legs each with inner row of spines on tibia and tarsus. Tibial spur formula 1,2,2, fore tibial spur very small.

*Genitalia*: Segment IX annular and short; acrotergite present dorsomesally. Preanal appendages short and rounded [21], setose. Rod-like process above tergum X absent. Tergum X membranous, undivided in dorsal view [6,10]; composed of a single elongated lobe, broad basally, rounded apically, slightly narrowed (Fig 11C). Inferior appendages broad basally, covered with setae; dorsal lobe broadly rounded, broad basally; ventral lobe absent; distal portion narrow, tapering posteriorly with apex acuminate (Fig 11D); small spine-like setae absent (Fig 11E). Phallic apparatus curved downward, large, rounded and inflated, occupying great part of segment IX length [9] (Fig 11F); phallic spine present, curved [9], helically, counter clockwise (Fig 11G).

*Material examined*: **Brazil**, **Bahia**, Curaçá, Recanto Campestre, Rio São Francisco, 08°59’56.7”S, 39°54’56.0”W, 357m, 04.v.2011, pan light trap U.V./white lights (França, D.)– 1 male, 8 females (alcohol); Riacho do Tio Zé, tributary of Rio Buracão, 09°07’48.1”S, 39°58’45.7”W, 362m, 05.v.2011, pan light trap U.V./white lights (França, D.)– 4 males, 21 females (alcohol); Pilão Arcado, Baixa do Brejo, 03.xii.2005, light trap U.V./white lights (Vieira, Alvim)– 1 male (alcohol); Lagoa do Morro, 26.iii.2006, light trap U.V./white lights (Alvim, Cordeiro)– 1 male, 2 females (alcohol); Buriti-Dunas, 04.x.2006, light trap U.V./white lights (Vieira, R.)– 1 male, 4 females (alcohol); **Paraíba**, Barra de Santana, Rio Paraíba, bridge BR-104, 07°31’44.3”S, 35°59’55”W, 336m, 31.vii.2009, pan light trap U.V./white lights (Calor, A.R., Lecci, L.S.)– 1 male, 5 females (alcohol); Areia, dam, 09°59’27.5”S, 35°45’06.5”W, 527m, 29.ix.2011, pan light trap U.V./white lights (Calor, A.R., Quinteiro, F.B., Gomes, V.)– 1 male (alcohol); **Pernambuco**, Afrânio, 08°31’53”S, 41°02’59”W, 550m., 16.v.2007, light trap U.V./white lights (Rafael, J.A., Xavier, F.F.)– 3 males, 9 females (pinned); **Piauí**, Caracol, Parque Nacional da Serra das Confusões, waterhole in Riacho dos Bois, 09°13’14.9”S, 43°29’20.4”W, 603m, 15.xii.2010, light trap U.V./white lights (França, D., Costa, AM.)– 3 males (pinned), 1 male (pinned; SEM); Santa spring, 09°13’8.8”S, 43°29’25.6”W, 558m, 11.xii.2010, light trap U.V./white lights (França, D., Costa, A.M)– 4 males, 3 females (pinned); same data except 14.xii.2010, light trap U.V./white lights (França, D., Costa, A.M)– 5 males, 6 females (pinned), 1 male (pinned/ SEM); **São Paulo**, São Carlos, córrego Fazzari, 21.iii.2007, light trap U.V./white lights (Lecci, L.S., Roque, F.O.)– 1 male (alcohol); Santa Rosa de Viterbo, Fazenda Águas Claras, 23.ix.2000, light trap U.V./white lights (Mendes, H.F., Andersen, T.)– 1 male (alcohol); Pedregulho, Furnas de São Pedro, 22.vi.2010 (Mateus, S., Lecci, L.S.)– 1 male, 1 female (alcohol).

*Distribution*: Bahamas, Bolivia, Canada, Colombia, Costa Rica, Cuba, El Salvador, Guatemala, Honduras, Jamaica, Mexico, Nicaragua, Panama, Peru, Puerto Rico, U.S.A., Venezuela, Brazil [BA, MG, PB, **PE (new record)**, PI, PR, SP].

*Taxonomic remarks*: Diagnostic characters for this species are discussed in the section of *O*. *excisa*. Ross [10] stated that the position of the crossvein *m-cu* in the forewing of this species is variable. The examined material showed two degrees of this variation: *m-cu* reaching fork V and *m-cu* reaching Cu_1a_ (after branching of Cu_1_ into Cu_1a_ and Cu_1b_) and that could be evidence that *Oecetis inconspicua* is a complex of species. Zhou et al. [63] and Floyd [64], based on molecular and larval data respectively, stated that the *inconspicua* group needs revision and there may be more than one species under the same name. *Oecetis inconspicua* has distribution records from south of Canada to south of Brazil, which is very unusual for caddisflies. This may be another evidence for a complex of species. A thorough examination on the *inconspicua* group like that made by Blahnik and Holzenthal on the *avara* group [5] is necessary.

*Oecetis paranensis* Flint, 1982

(Fig 12)

**Fig 12:**
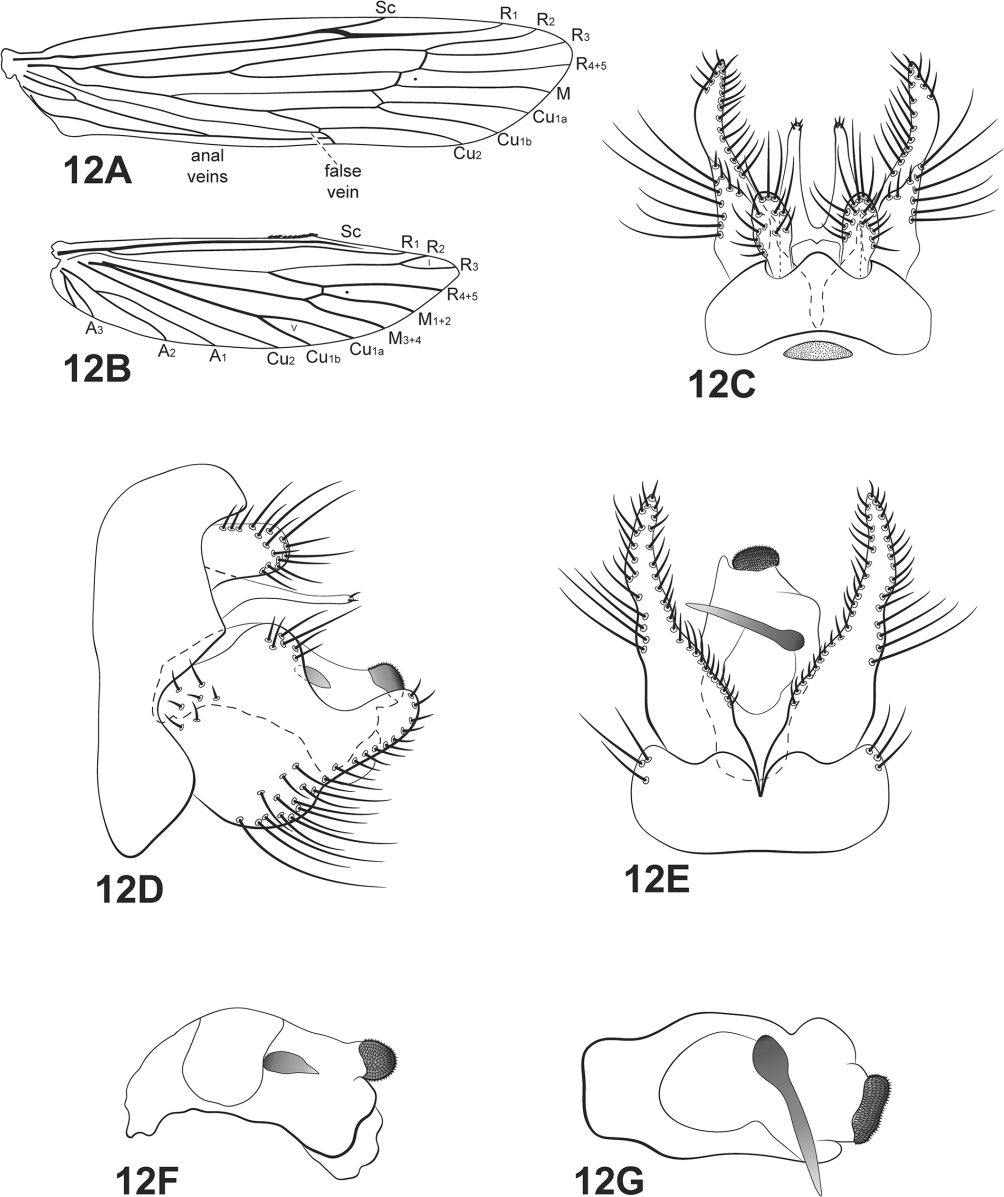
*Oecetis paranensis* Flint. A, forewing. B, hind wing. C, male genitalia, dorsal view. D, male genitalia, lateral view. E, male genitalia, ventral view. F, phallic apparatus, lateral view. G, phallic apparatus, dorsal view. Abbreviation of the wing structures: Sc, subcosta; R, radius; M, medius; A, anal vein; I, fork 1; V, fork 5.

*Oecetis paranensis* Flint, 1982 [32]: 46.

*Male*: body length 6.2 (n = 6). Forewing length 6–9 mm [32].

Head: color brown [32]. Antennae long, 3 times length of forewing; scape and pedicel both short and stout, about same length as the flagellar segments. Maxillary palps brown, 5-segmented, all segments subequal in length and width, densely covered with setae. Labial palps brown, 3-segmented, first segment very reduced.

Thorax: pterothorax brown in dorsal view and yellow brownish in lateral and ventral views. Forewings with golden-brown setae [32]; dark bands over cord absent; dark spots on forks, junctions and end of veins [32]; *m-cu* crossvein reaching Cu_1a_ (after branching of Cu into Cu_1a_ and Cu_1b_) (Fig 12A). Hind wings with forks I and V [32]; basal brush present (Fig 12B). Legs yellowish brown, mid and hind legs each with inner row of small spines on tarsus. Tibial spur formula 1,2,2, fore tibial spur very small.

*Genitalia*: Segment IX annular and short [32]; one acrotergite present, dorsomesally. Preanal appendages short and rounded [32], with setae. Tergum X membranous, deeply divided mesally, forming two lobes, apex with small setae in dorsal view [9,32] (Fig 12C). Inferior appendages broad basally, covered with setae; dorsal lobe broad and rounded [32]; ventral lobe broader than dorsal lobe and rounded; distal portion short and narrow [32], apex rounded (Fig 12D); small spine-like setae absent (Fig 12E). Phallic apparatus, in lateral view, slightly curved downward, short, straight with ventral elongated tip [32], constricted in middle line (Fig 12F); phallic spine present [32], straight; phallotremal sclerite U-shaped and a small membranous lobe capped by a cluster of spicules on tip [32] (Fig 12G).

*Material examined*: **Brazil**, **Bahia**, Curaçá, 08°59’57.6”S, 39°54’47.2”W, 06.v.2011, light trap U.V./white lights (Silva-Neto, A.M.)– 2 males, 1 female (alcohol); Recanto Campestre, Rio São Francisco, 08°59’56.7”S, 39°54’56.0”W, 357m, 06.v.2011, pan light trap U.V./white lights (França, D.)– 1 male, 1 female (alcohol); Pilão Arcado, Buriti-Dunas, 04.x.2006, light trap U.V./white lights (Vieira, R.)– 1 male, 2 females (alcohol); Ilhéus, around Parque Estadual da Serra do Conduru, farm, 14°28’12.6”S, 39°04’41.1”W, 04.vii.2010, light trap U.V./white lights (Calor, A.R., Quinteiro, F.B., França, D., Mariano, R.)– 1 male (pinned); **São Paulo**, Ribeirão Preto, *campus* USP, Lago Monte Alegre, 14.viii.2007, pan light trap U.V./white lights (Calor, A.R., Mariano, R., Pinho, L.C., Moretto, R.A.)– 3 males, 5 females (alcohol).

*Distribution*: Argentina, Bolivia, Paraguay, Peru, Brazil [BA, MG, **SP (new record)**].

*Taxonomic remarks*: the description provided by Flint matches the specimens examined, although the colors have small differences when comparing dry and alcohol preserved specimens. This is especially notable in the forewing setae. They tend to fall off in alcohol, so it was not possible to see the golden brown setae and the dark setae over the forks, junctions and ends of veins.

This species is diagnosed from its congeners by the shape of tergum X, inferior appendage and the phallic apparatus. Tergum X is divided basally, forming two terete processes with digitate apices. The only similar species is *Oecetis oberdorffi* Rueda-Martín, Gibon, Molina [6], which has an inferior appendage similar to *O*. *inconspicua*, with a C-shaped incision between the thumb-like dorsal lobe and the elongated and rounded distal lobe. The inferior appendage of *Oecetis paranensis* has a broad dorsal lobe and a digitate distal lobe, with an almost 90° angle between them. Also, *O*. *paranensis* has a broad ventral lobe on the inferior appendage, which is absent in *O*. *oberdorffi*. Finally, *O*. *paranensis* has a character that no other *Oecetis* has: a cluster of spicules on the tip of the endotheca.

*Oecetis punctipennis* (Ulmer, 1905)

(Figs 13 and 9D)

**Fig 13:**
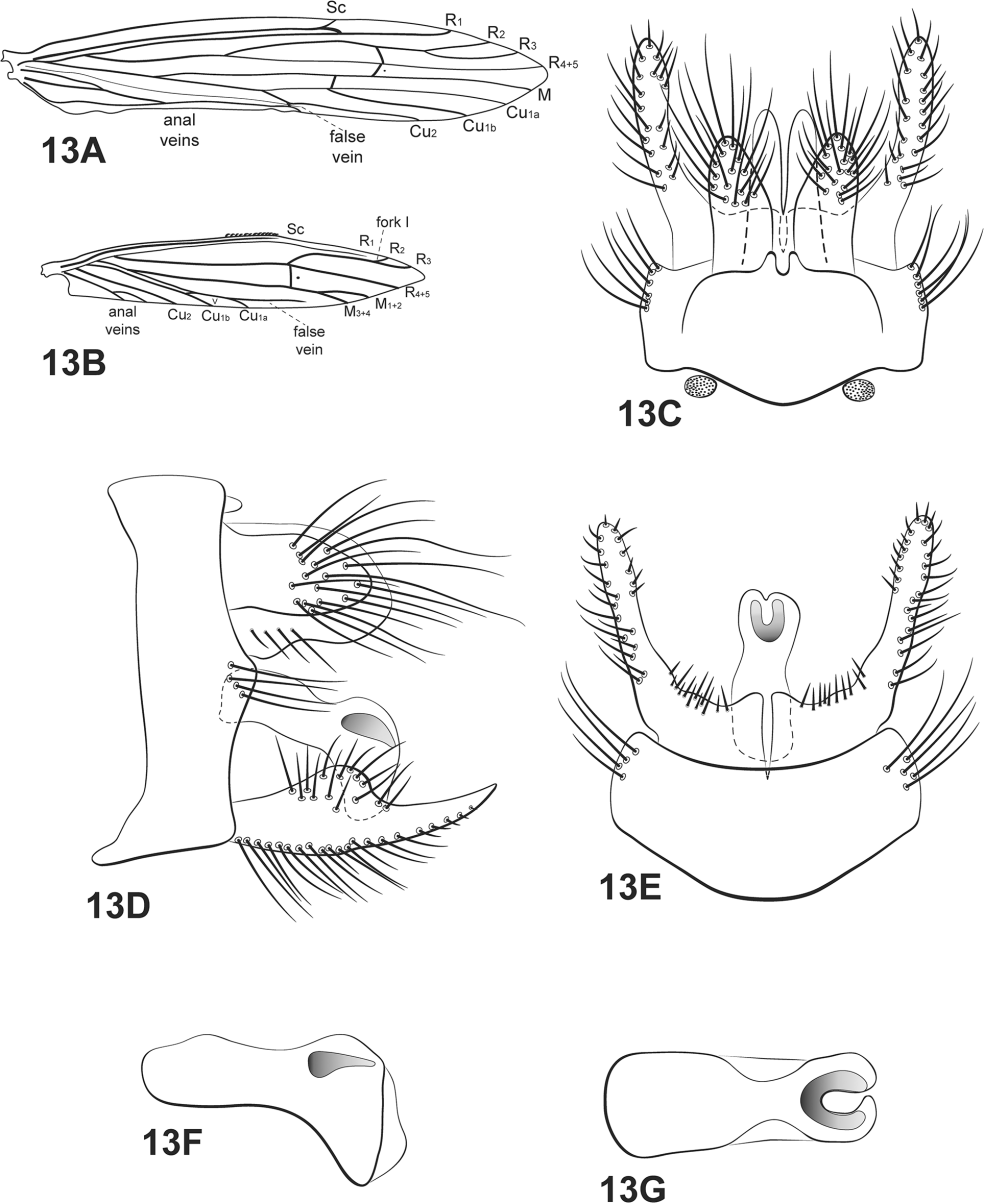
*Oecetis punctipennis* (Ulmer). A, forewing. B, hind wing. C, male genitalia, dorsal view. D, male genitalia, lateral view. E, male genitalia, ventral view. F, phallic apparatus, lateral view. G, phallic apparatus, dorsal view. Abbreviation of the wing structures: Sc, subcosta; R, radius; M, medius; A, anal vein; I, fork 1; V, fork 5.

*Pseudosetodes punctipennis* Ulmer, 1905 [13]: 77

*Oecetina parishi* Banks, 1914: 631 [65] (male); Flint, 1966: 10 [15] (to synonymy).

*Oecetis bridarollina* Navás, 1933 [66] (male); Flint, 1972: 245 [52] (to synonymy).

*Male*: body length 5.4 mm (n = 12). Forewing length 5–8 mm [23].

Head: color pale yellow [13]. Antennae long, about 3.5 times length of forewings; scape and pedicel both short and stout, about same length as the flagellar segments. Maxillary palps yellow, covered with brown setae [13]; 5-segmented, all segments subequal in length and width. Labial palps pale yellow, 3-segmented, first segment very small.

Thorax: pterothorax yellow dorsally, scutellum brown [13]; pale yellow in ventral view [13]. Forewings yellowish brown, hyaline [13]; dark bands absent; dark spots on forks, junctions and end of veins [13,23]; *m-cu* crossvein reaching Cu (before branching into Cu_1a_ and Cu_1b_), or fork V (Fig 13A). Hind wings with forks I and V; basal brush present (Fig 13B). Legs light yellow [13]; mid legs each with row of spines on tibia and tarsus; hind legs each with row of spines on tarsus; tibial spur formula 1,2,2; apical spur of fore tibia very small [13].

*Genitalia*: Segment IX annular and short; two small acrotergites present dorsolaterally. Preanal appendages long, broad, with apex rounded, setose. Rod-like process above tergum X absent. Tergum X, in dorsal view (Fig 13C), membranous, divided posteriorly [9]. Inferior appendages broad basally, covered with setae [9]; dorsal lobe slightly rounded; ventral lobe absent; distal portion narrow, tapering posteriorly with apex acute [23] (Fig 13D); small spine-like setae absent (Fig 13E). Phallic apparatus, in lateral view (Fig 13F), curved downward, short [9], comma shaped with ventral elongated tip, constricted in middle line [9]; phallic spine absent [9]; phallotremal sclerite U-shaped (Fig 13G).

*Material examined*: **Brazil**, **Bahia**, Wenceslau Guimarães, Estação Ecológica Estadual Wenceslau Guimarães, Riacho Serra Grande, 13°35’43”S, 38°43’12”W, 531m, 08.x.2010, pan light trap U.V./white lights (Calor, A.R., Quinteiro, F.B., e col.)– 5 males, 2 females (alcohol); 13°35’34”S, 39°42’52”W, 482m, 09.x.2010 – 1 male (alcohol); same data except 10.x.2010 – 1 male, 1 female (alcohol); Mata de São João, Reserva Sapiranga, 12°33’41.7”S, 38°02’42.9”W, 22-25.vii.2001, Malaise trap (Tavares, M.T.*et al*.)– 3 males (alcohol); Senhor do Bonfim, Serra Santana, 28.xi.2006 (Souza, Monteiro, Alvim, Zacca)– 2 males (alcohol); Lençóis, Parque Nacional da Chapada Diamantina, Rio Santo Antônio, 12°29’57.9”S, 41°19’75.2”W, 340m, 26.x.2008, light trap U.V./white lights (Calor, A.R., Mariano, R., Mateus, S.)– 5 males (pinned), 1 male (Pinned; SEM); **Ceará**, Barbalha, Geosítio Riacho do Meio, Riacho do Meio, 07°21’59.4”S, 39°19’48.8”W, 04.ii.2011, light trap U.V./white lights (Quinteiro, F.B, Costa, A.M.)– 1 male (alcohol), 2 males (pinned), 1 male (pinned/ SEM); Distrito de Arajara, Arajara Park, Gruta do Farias, 24.vii.2009, light trap U.V./white lights (Calor, A.R., Lecci, L.S.)– 3 males (alcohol).

*Distribution*: Argentina, Bolivia, Costa Rica, Ecuador, Guyana, Nicaragua, Panama, Peru, Surinam, Venezuela, Brazil [BA, CE, MG, PA, PE RJ, SP].

*Taxonomic remarks*: see remarks for *O*. *iguazu*.

### Key to males of *Oecetis* from Brazil

The dichotomous key is based primarily on male genitalic characters for species recorded from Brazil. Since some species are difficult to diagnose, it is recommended that use of the key be complemented with the synopses, illustrations, and original descriptions.

1. Phallic apparatus with phallic spine (Figs 5F, 6F, 8F and 11F)....................2

Phallic apparatus without phallic spine (Figs 1 and 4F, 7F, 10F, 13F)....................9

2(1). Inferior appendages elongate, tapering distally (Figs 5D, 8D and 11D)....................3

Inferior appendages short (Figs 2D, 6D and 12D)....................6

3(2). Phallic apparatus enlarged in width, rounded and inflated; tergum X not divided (sometimes with only a small incision apically) (Fig 8F and 8G, 11F and 11G; 8C and 11C)....................4

Phallic apparatus not as above; tergum X divided mesally (Fig 5C, 5F and 5G)....................5

4(3). Dorsal lobe on inferior appendages enlarged basally (Fig 11D and 11E)....................***Oecetis inconspicua*** (Walker) [21]

Dorsal lobe on inferior appendages thumb-like (not enlarged basally) (Fig 8E)....................***Oecetis excisa*** Ulmer [19]

5(3). Tergum X apex, in lateral view, broad and rounded; forewing without dark spots; segment IX with a pair of dorsolateral processes; rod-like process above tergum X present (Fig 5C and 5D)....................***Oecetis froehlichi* sp. nov.**

Tergum X apex, in lateral view, narrow and acuminate; forewing with dark spots on cord and end of the majority of the veins; segment IX without a pair of dorsolateral processes; rod-like process above tergum X absent (Flint [18] Fig 273)....................***Oecetis doesburgi*** Flint [18]

6(2). Phallic spine straight (Fig 12E and 12G)....................7

Phallic spine curved (Fig 6F and 6G)....................8

7(6). Segment IX with a pair of dorsolateral processes; rod-like process above tergum X present; two or more phallic spines in the phallic apparatus; tergum X divided with a median incision, forming two broad lobes on base and tapering apically (Henriques-Oliveira *et al*., [8], Fig 2B–2D)....................***Oecetis danielae*** Henriques-Oliveira, Dumas & Nessimian [8]

Segment IX without a pair of dorsolateral processes; rod-like process above tergum X absent; only one phallic spine in the phallic apparatus; tergum X divided from its base into two digitate projections (Fig 12C–12E)....................***Oecetis paranensis*** Flint [23]

8(6). Tergum X apex, in lateral view, narrow and acuminate; forewing with dark spots on cord and end of the majority of the veins; sternum IX with a distinct cylindrical posterior projection (Henriques-Oliveira et al., [8], Fig 3A and 3B and 3D).................... ***Oecetis iara*** Henriques-Oliveira, Dumas & Nessimian [8]

Tergum X apex, in lateral view, broad and rounded; forewing without dark spots; sternum IX not as above (Fig 6A, 6D and 6E)....................***Oecetis amazonica*** (Banks) [17]

9(1). Phallic apparatus comma shaped, with ventral apex projecting and strongly down turned (Figs 7F, 10F and 13F)....................10

Phallic apparatus not as above (Figs 1F–4F)....................14

10(9). Preanal appendages fused with tergum X; hind wing venation reduced (Cu_1_ unbranched and small anal area) (Fig 7A–7D)....................***Oecetis connata*** Flint [18]

Preanal appendages not fused with tergum X; hind wing venation usual for the genus (Cu_1_ branches into Cu_1a_ and Cu_1b_) (Fig 10C and 10D, 13C and 13D; 10B and 13B)....................11

11(10). Tergum X shorter than the preanal appendages (Rueda-Martín *et al*., [6], Fig 5B and 5C, 8B and 8C)....................12

Tergum X as long as or longer than the preanal appendages (Fig 10C and 10D, 13C and 13D)....................13

12(11). Inferior appendages short and quadrate; forewing with dark spots on cord and end of the majority of the veins; tergum X very reduced and barely noticeable (Flint [22], Fig 145; Rueda-Martín *et al*., [6], Fig 8A–8C)....................***Oecetis knutsoni*** Flint [22]

Inferior appendages elongate, tapering distally; forewing without dark spots; tergum X well developed (Rueda-Martín *et al*., [6], Fig 5A–5C)....................***Oecetis dominguezi*** Martin, Gibon & Molina [6]

13(11). Inferior appendages without dorsal lobe (Fig 10D)....................***Oecetis iguazu*** Flint [20]

Inferior appendages with dorsal lobe (Fig 13D)....................***Oecetis punctipennis*** (Ulmer) [13]

14(9). Segment IX with a pair of dorsolateral processes (Fig 1C and 1D, 4C and 4D)....................15

Segment IX without a pair of dorsolateral processes (Fig 2C and 2D, 3C and 3D)....................18

15(14). Inferior appendages with dorsal lobe (Fig 4D)....................16

Inferior appendages without dorsal lobe (Fig 1D)....................17

16(15). Dorsolateral processes on segment IX divided into ventral and dorsal lobes; dorsal lobe on inferior appendages cylindrical and terete (Fig 4D)....................***Oecetis furcata* sp. nov.**

Dorsolateral processes on segment IX undivided; dorsal lobe on inferior appendages small and subtle (Quinteiro & Calor [7], Fig 1D)....................***Oecetis fibra*** Chen & Morse [7]

17(15). Tergum X as long as the preanal appendages; rod-like process above tergum X present; dorsolateral process on segment IX bent ventrally and enlarged in width on 2/3 of its length (Fig 1C and 1D)....................***Oecetis achantostema* sp. nov.**

Tergum X longer than the preanal appendages; rod-like process above tergum X absent; dorsolateral process on segment IX straight, cylindrical and tapering apically (Flint [24], Figs 13–15)....................***Oecetis rafaeli*** Flint [24]

18(14). Inferior appendages elongated, tapering distally; rod-like process above tergum X present; fringe of long setae on the base of M vein forewing (internal surface) present (Fig 3A–3D)....................***Oecetis clavicornia* sp. nov.**

Inferior appendages short; rod-like process above tergum X absent; fringe of setae on the base of M vein forewing absent (Fig 2A–2E)....................19

19(18). Apex of tergum X rounded, in dorsal view; tergum X shorter than preanal appendages; forewing without dark spots (Henriques-Oliveira *et al*., [8], Fig 1A–1C)....................***Oecetis angelae*** Henriques-Oliveira, Dumas & Nessimian [8]

Apex of tergum X acute, in dorsal view; tergum X as long as, or longer than preanal appendages; forewing with dark spots on cord and ends of the majority of the veins (Fig 2A and 2C)....................***Oecetis martinae* sp. nov.**

## Forewing Venation Discussion

It is traditionally accepted that the M vein in Trichoptera branches into M_1+2_ and M_3+4_ similarly to the Cu_1_ vein (presence of fork V) [38]. In this way, the forewing venation in caddisflies is considered to be fairly simple and the variations differing from the cited pattern always constitute simplifications [38]. However, it is possible to observe that other more distal branching events can occur and the order is filled with examples of different degrees of variation in M and Cu_1_ branching. But all of them follow the above rule of branching in which M branches into M_1+2_ and M_3+4_ and sometimes they branch even more.

In some genera, such as *Molanna* Curtis [67] or *Rhyacophila* Pictet [68], M_1+2_ and M_3+4_ also branch into M_1_, M_2_, M_3_ and M_4_ (presence of forks III and IV). Also, there are intermediary forms, such as taxa in which M_1+2_ branches and M_3+4_ does not (presence of fork III only), for example, species of the genus *Ithytrichia* Eaton [69].

Usually, genera in Trichoptera have a well defined hypothesis of vein homology not requiring a discussion. However, two different interpretations of forewing venation have been used in *Oecetis* with no consensus reached. The two hypothesis are: 1. “M vein branches” (supported by [45,59,70], for example); and 2. “M vein does not branch” (supported by [4,71,72], for example). The first approach postulates that the M vein in the forewing branches into M_1+2_ and M_3+4_, while the second postulates that the M vein remains unbranched until the end of the wing.

The unbranched M vein hypothesis was first proposed by McLachlan [4] who used this character as diagnostic for the genus *Oecetis*. No direct reference to a different hypothesis was made until Betten [59] thoroughly discussed venation hypothesis for many species of caddisflies, including *Oecetis*. Betten [59] pointed out that the unbranched condition of the M vein in *Oecetis* was only a misinterpretation, and Ulmer [71] and McLachlan [4] were mistaken in describing the M vein as unbranched.

Both hypotheses have been followed by different authors and no further discussion about their validity has been raised lately. This makes the wings hard to compare due the absence of consensual homology hypothesis among the veins. Therefore, this condition could lead to mistakes when describing new species or proposing phylogenetic hypothesis, for example. For this reason, we argue in favor of a standardized *Oecetis* wing nomenclature, based on a consistent homology hypothesis. We present evidence that an unbranched M vein constitutes a better homology interpretation.

Observing the positions of the *m-cu* crossvein in some *Oecetis* species, such as *O*. *arcada* Mosely [73], *O*. *laustra* Mosely [73], *O*. *parallela* Wells [74] and *O*. *pseudoamazonica* Rueda-Martín, Gibon and Molina [6], it is noticeable that the crossvein varies in position along the Cu vein. Sometimes it reaches Cu vein anterior to the fork, other it intersects posterior to the fork, and, in a third way, it intersects the Cu vein at the fork. The last pattern is the most common in *Oecetis*, as in *O*. *excisa* Ulmer [19], and may lead the false impression that M vein branches and Cu does not. This variation in the position of the *m-cu* crossvein may be seen even within a single species, as pointed by Ross ([10], p. 243) for *O*. *inconspicua*: “*Position of crossveins forming the cord extremely variable*, *ranging from a condition in which the three crossveins form an almost straight line to one in which they are far removed and steplike*.”

Comparing the M and Cu_1_ branching patterns in Leptoceridae (under the most recent phylogenetic hypothesis [2]), it is noticeable that in all of them the fork V is present, *i*.*e*. the Cu_1_ vein branches in all cases in Cu_1a_ and Cu_1b_ and that applies also to *Oecetis*. If the “M vein branches” hypothesis is accepted, we would have to assume that the Cu_1_ does not branch in *Oecetis*, what seems very unlikely to happen observing the wing patterns in leptocerids.

Yet, it would be possible to assume that the M vein branches into M_1+2_ and M_3+4_ and the Cu_1_ branches into Cu_1a_ and Cu_1b_. If so, we would have to assume that the Cu_2_ would be fused to the anal veins (as Betten [59] does) which seems to be very unlikely due to the often clear separation of the anal area in most of the caddisfly families.

At last, it can be considered that if we assume the false vein to be interpreted as Cu_2_, we could use a hypothesis in which Cu_1_ branches and the M vein can be interpreted as also branched. However, we consider that the false vein is an artifact of the convoluted membrane, as described by Morse [70]. When observing the specimens, it is possible to notice that the false vein has a distinct texture and it is usually much thinner than any other vein on the wings. It leads us to believe that the false vein is not a “true” vein and that is the reason we exclude this vein from our discussion and, as a consequence, do not considerate this hypothesis with both Cu_1_ and M branching.

Considering the evidences listed above, it is possible to indentify which one is the *m-cu* crossvein in *Oecetis*. With that in mind, we assume that the hypothesis in which M does not branch, seems to be the most reasonable one.

Standardizing a forewing nomenclature will make all homology hypotheses comparable to each other, as well as to provide a single framework for phylogenetic hypothesis within Leptoceridae and *Oecetis*.

It is understandable that many other researchers will still support the branched M vein hypothesis. In this way, more accurate studies, such as developmental biology, remain useful in this area to provide stronger evidence in this subject.
